# Leveraging epigenetic aberrations in the pathogenesis of endometriosis: from DNA methylation to non-coding RNAs

**DOI:** 10.3389/fgene.2025.1597287

**Published:** 2025-07-28

**Authors:** Hajar Erraji, Adil El Ghanmi, Noureddine Louanjli, Mohamed Benahmed, Fadoua El Mansouri, Mohammed Zarqaoui, Bouchra Ghazi

**Affiliations:** ^1^ Immunopathology-Immunotherapy-Immunomonitoring Laboratory, Faculty of Medicine, Mohammed VI University of Sciences and Health (UM6SS), Casablanca, Morocco; ^2^ Reproductive Health Physiopathology Laboratory, Mohammed VI Center for Research and Innovation (CM6RI), Rabat, Morocco; ^3^ Department of Obstetrics and Gynecology, Mohammed VI International University Hospital, Bouskoura, Morocco; ^4^ Laboratory of Medical Analysis and Reproductive Biology, Labomac, Casablanca, Morocco; ^5^ Department of Obstetrics and Gynecology, Les Iris Clinic, Casablanca, Morocco; ^6^ National Institute of Health and Medical Research (INSERM), Mediterranean Center for Molecular Medicine (C3M), Nice, France

**Keywords:** endometriosis, epigenetic modifications, DNA methylation, histone modifications, non-coding RNAs, micro-RNAs, long-RNAs, infertility

## Abstract

Endometriosis is highly underdiagnosed and undertreated gynecological disorder, with diagnosis often delayed by 8–12 years. This delay can have serious consequences including infertility. Currently, the gold standard for endometriosis diagnosis and treatment is laparoscopy, an invasive surgical intervention. The molecular mechanisms underlying the onset of endometriosis are yet unclear, but it is assumed that epigenetic modifications are an important contributor in the etiopathology of the disease. Given that, dissecting the features of epigenetic aberrations underlying endometriosis can be a crucial step toward developing early and accurate non-invasive diagnostic tools. Accurate and timely diagnosis of endometriosis can significantly reduce healthcare costs, and enhance women’s social wellbeing. Epigenetic modifications especially DNA methylation, micro-RNAs and long-RNAs, hold promise as potential biomarkers for the early diagnosis of endometriosis. This review underscores the innovative potential of epigenetic mechanisms as early biomarkers for endometriosis diagnosis. We summarize and critically discuss recent findings and epigenetic modifications role in endometriosis pathophysiology, from DNA methylation and histone modifications to non-coding RNAs in different tissues.

## 1 Introduction

**FIGURE 1:**
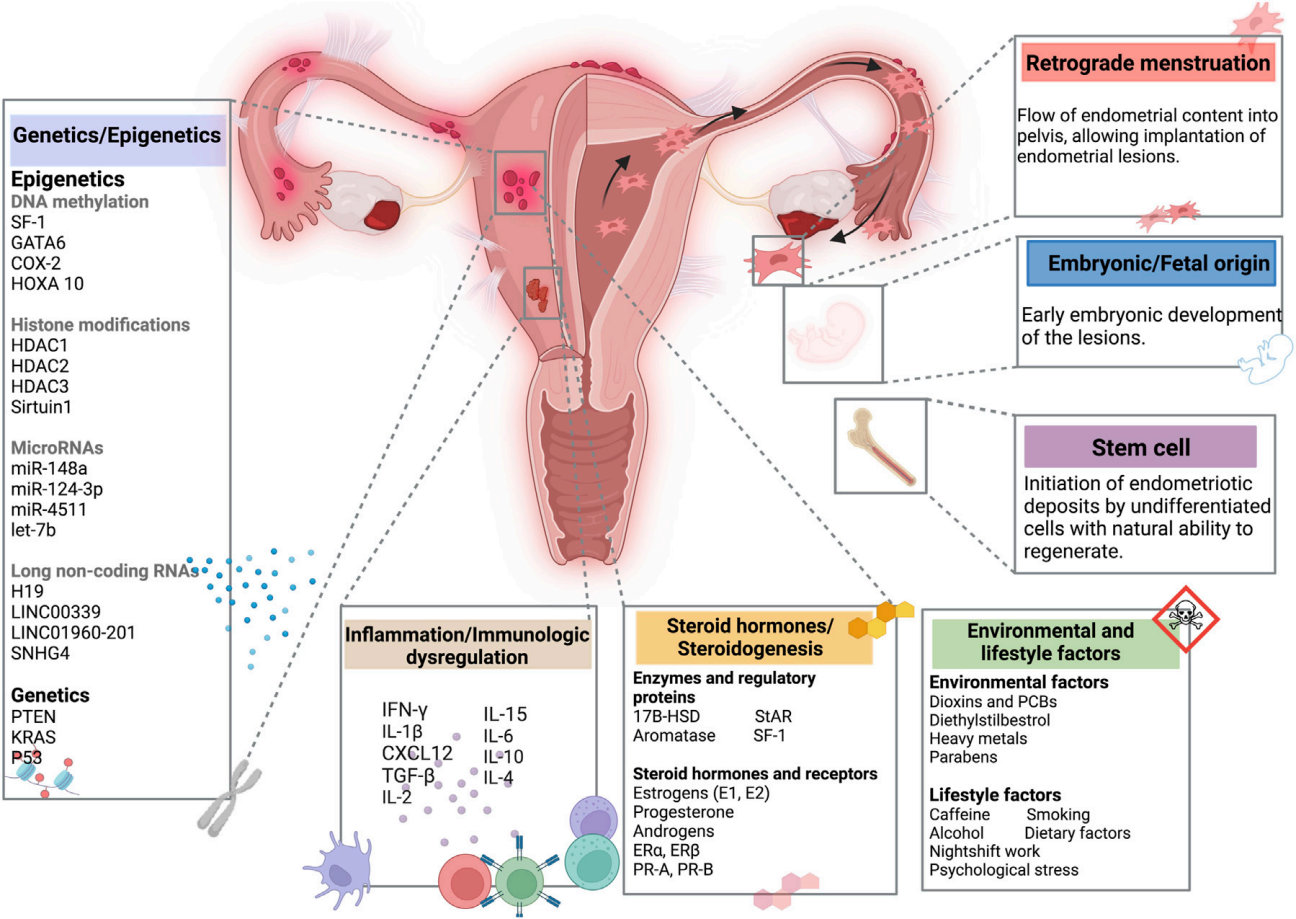
Summary of the pathogenesis of endometriosis. Created with BioRender (https://www.biorender.com/).

Endometriosis is a benign gynecological pathology defined by the presence of endometrial tissue outside the uterine cavity (Taylor et al., 2021). Patients with endometriosis can be asymptomatic, while others can have symptoms of dyspareunia, dysmenorrhea, irregular uterine bleeding, and chronic pelvic pain (Parasar et al., 2017; Taylor et al., 2021). This debilitating disease occurs in nearly 10% of women of reproductive age, being one of the main reasons of subfertility or infertility in women (Skorupskaite and Bhandari, 2024). Numerous theories, such as coelomic metaplasia, implantation, or embryonic stem cells, have been proposed to explain the pathophysiology of endometriosis, even though the disease’s cause is yet unknown (Mariadas et al., 2025). Figure 1 shows the set of theories related to pathogenesis of endometriosis. Indeed, the most established theory is that endometrial tissue seeds in ectopic locations as a result of retrograde menstruation, which may be connected to hematogenous or lymphatic circulation (Adilbayeva and Kunz, 2024). Therefore, pelvic implantation and durability are influenced by additional hormonal or immunological-related variables (Parasar et al., 2017). Even though retrograde menstruation is very common, endometriosis appears only in some women presenting with specific cellular and molecular features in peritoneal or eutopic endometrial tissue (Lucidi et al., 2005; Bulun, 2009; Mariadas et al., 2025). Endometriosis occurs due to specific genetic, epigenetic, environmental and immune factors (Mariadas et al., 2025). It is important to note that between 25% and 50% of patients with infertility have endometriosis (Mathyk et al., 2024). Although the relationship between endometriosis and infertility is still up for debate, their connection is clinically acknowledged and has strong evidence in the literature (Bonavina and Taylor, 2022). Right now, endometriosis-associated infertility is considered to be a multifactorial disorder, faced with challenges related to immune, genetic and epigenetic alterations affecting not only the integrity of fallopian tubes and embryo migration, but also the endometrium receptivity and embryo implantation (Macer and Taylor, 2012). Infertility in all forms of endometriosis can be caused by impaired folliculogenesis, low quality of oocytes, ovulation disturbances, aberrant embryogenesis, or an impaired implantation process (Qi et al., 2025). On that account, the pathological process of infertility in endometriosis is complex and represents one of the serious consequences of delayed diagnosis with an average time of 6–12 years (Beloshevski et al., 2024). Both clinical and social factors are accountable for this delay, resulting in compounding financial, emotional and physical burdens for women (Kocas et al., 2023).

According to reports, many women put off getting help for endometriosis symptoms because they feel embarrassed talking about period pain and menstrual irregularities, fear of stigmatization, or believe their doctor did not treat their symptoms seriously (Kocas et al., 2023). As previously mentioned, symptoms of endometriosis are often similar to those of other pelvic conditions. This similarity mandates healthcare professionals to enhance their clinical vigilance and expertise in order to ensure a prompt diagnosis. The gold standard for endometriosis diagnosis is typically laparoscopy, a surgical procedure offering numerous advantages over traditional open surgery (Simko and Wright, 2022). In the context of endometriosis, the advantage of laparoscopy is that it is both diagnostic and therapeutic, being the state of the art treatment for endometriosis that reduces pain (Bafort et al., 2020). Despite the effectiveness of laparoscopy, it is considered an invasive diagnostic tool with many limitations such as general anesthesia, and cost considerations (Simko and Wright, 2022). Furthermore, endometriosis frequently recurs, with nearly 50% of women requiring additional intervention within 5 years (Saunders and Horne, 2021). This underscores the importance of identifying reliable non-invasive biomarkers for endometriosis detection at an early stage, in order to minimize the frequency of laparoscopic surgery without compromising patient clinical outcomes (Kaspute et al., 2024). Recent research has focused on epigenetic mechanisms, considering the fundamental role of estrogen and progesterone in regulating cellular processes during the endometrial cycle (Yang et al., 2023; Yu et al., 2024; Mariadas et al., 2025). These processes are also linked to particular transcriptional profiles that are essential for normal endometrial function (Retis-Resendiz et al., 2021). Epigenetics is generally defined as heritable changes in gene expression without altering the DNA sequence (Felsenfeld, 2014). It is associated with fundamental processes such as cellular identity, development and homeostasis (Rodenhiser and Mann, 2006; Blakey and Litt, 2015). Epigenetics include DNA methylation, histone post-translational modifications, and non-coding RNAs (Shu et al., 2023). This review intends to present an overview of the ever-growing recent evidence of epigenetic contributions in endometriosis pathophysiology, with particular emphasis on DNA methylation, histone modifications, and non-coding RNAs. The impact of epigenetic modifications on endometriosis-related immune events and infertility are also discussed.

## 2 Epigenetic of endometriosis

Epigenetics defines the study of molecular alterations in chromatin that control gene expression and maintain genome stability, without altering the DNA sequence (Kumari et al., 2022). Through the regulation of DNA folding, chromatin compaction, nuclear arrangement, and transcript stability, these processes influence gene expression (Hsiao et al., 2017; Martin and Fry, 2018). Epigenetics is one of the key factors controlling cellular differentiation and determining cell phenotype (Meissner et al., 2008). It plays a critical role in maintaining the correct, undisturbed development of the organism (Kumari et al., 2022). A complex epigenetic patterns emerges when epigenetic changes occur at the wrong time or in the wrong place leading to the development of many complex human diseases (Esteller, 2002). Numerous studies have highlited the epigenetic contribution to the pathogenesis of endometriosis (Hsiao et al., 2017; Bedrick et al., 2024). It is noteworthy that the epigenome is dynamically regulated by the interplay of environmental factors, hormonal status, and immune microenvironment (Bulun et al., 2019). Epigenetic modifications include DNA methylation, histone modifications, as well as non-coding RNAs (Shu et al., 2023). Because of their dynamic and changeable nature, epigenetic modifications hold the potential to be used as early biomarkers and therapeutic tools (Dai et al., 2024).

### 2.1 DNA methylation

DNA methylation is an epigenetic chromatin mark that allows heterochromatin formation, gene silencing, and regulates alternative splicing. Exons have higher levels of DNA methylation compared to flanking introns. Around 22% of alternative exons splicing is regulated by DNA methylation (Lev Maor et al., 2015). Two mechanisms use DNA methylation to regulate alternative splicing. The first one involves modulation of the elongation rate of RNA polymerase II, and the second one involves heterochromatin protein 1 protein, a fundamental unit of heterochromatin packaging, that recruits splicing factors onto transcribed alternative exons (Pappalardo and Barra, 2021).

DNA methylation is one of the most common epigenetic modifications, regulating gene expression by recruiting repressive proteins or through the inhibition of transcription factor binding (Moore et al., 2012). It consists of adding a methyl group to the fifth position of cytosine in CpG sites (Moore et al., 2012). DNA methylation can occur in different genomic regions namely, Intergenic Regions, Promoters, Gene Body and Enhancers (Moore et al., 2012; Kreibich et al., 2023). DNA methyltransferases (DNMTs) carry out this process, by using S-adenosylmethionine as the methyl donor to catalyze the addition of the methyl group to the cytosine ring to generate methyl cytosine (Gao et al., 2018) DNA methylation is a dynamic process that requires *de novo* DNA methyltransferases DNMT3A and DNMT3B, involved in adding methyl groups to cytosine at unmethylated DNA (Tóth et al., 2025). The next step consists of preserving the novel methylation patterns by DNMT1 (Tóth et al., 2025). During DNA replication, DNMT1 enzyme is recruited to ensure the inherence of the parental methylation pattern in the newly synthesized strands (Xu et al., 2025). The silencing achieved through the methylation at CpG sites can directly block transcription factor binding due to the methylation of response elements (Moore et al., 2012). Furthermore, another mechanism of gene regulation by DNA methylation involves the methyl-CpG-binding domain (MBD) protein MeCP2, MBD1, MBD2, and MBD3 (Wood and Zhou, 2016). These proteins bind to methylated DNA and recruit corepressor complexes, including histone deacetylases (HDACs), making the DNA less accessible for transcription (Newell-Price et al., 2000; Javaid and Choi, 2017). This leads to a stable transcriptional repression of the target genes (Miller and Grant, 2013). Beyond the classical dogma, there is growing evidence of a more complex effect of DNA hypermethylation on gene expression depending of the biological context. For instance, the expression of hypermethylated genes can be unaffected or even upregulated. Furthermore, some transcription factors tend to bind methylated rather than unmethylated CpGs (Rauluseviciute et al., 2020).

In endometriosis, there are more than 40,000 CpG sites, distal to classical CpG islands, differentially methylated (Dyson et al., 2014; Gerkowicz et al., 2020; Zubrzycka et al., 2020; Adamczyk et al., 2022). Furthermore, it has been proven that the expression patterns of DNMTs in endometriotic tissue differ from those of normal endometrium (Wu et al., 2007; Hsiao et al., 2015; Zubrzycka et al., 2020). Regarding DNA methylation, altered expression of DNMT1, DNMT3A and DNMT3B was shown in ectopic endometrium, compared to normal controls and eutopic endometrium of women with endometriosis (Wu et al., 2007).

#### 2.1.1 Hypomethylation

The accurate regulation of DNA methylation profiles is crucial to cell function and normal development of adult organisms (Meng et al., 2024). DNA methylation stability depends on the cooperation between *de novo* DNA methyltransferases, Dnmt3A and Dnmt3B, Dnmt3L, microRNAs, lymphoid-specific helicase (Lsh) and other factors (Pogribny and Beland, 2009). Disruption of any of these factors can lead to alteration of the normal methylation state, leading to DNA hypomethylation (Pogribny and Beland, 2009). DNA hypomethylation involves several pathways and can be achieved through passive or active mechanisms (Liu J. et al., 2022). Passive demethylation of the genome can results of limited availability of the universal methyl donor S-adenosyl-l-methionine (SAM), compromised integrity of DNA, and altered expression and/or activity of DNA methyltransferases (DNMTs) (Pogribny and Rusyn, 2014). Regarding the active demethylation, it occurs independently of DNA replication, and could be achieved by removal of the base itself, removal of the methyl group, or by conversion of the base into an intermediate that could be resolved or replaced by unmodified cytosine (Bagci and Fisher, 2013) The DNA repair machinery, precisely and timely repair the DNA damage to maintain the genome integrity (Kadam et al., 2024). Interestingly, recent investigations have provided a connection between active DNA demethylation and the activity of DNA repair machinery (Schuermann et al., 2016).

It is well established that DNA hypomethylation plays a significant role in human carcinogenesis through different mechanisms, namely, activation of oncogenes, transposon reactivation, and inducing chromosomal instability (Molefi et al., 2025). DNA hypomethylation is also observed in other diseases such as cardiovascular (Krolevets et al., 2023), neurodegenerative (Daily et al., 2023), and gynecological diseases, notably endometriosis (Mortlock et al., 2023; Baldi et al., 2025; Hu et al., 2025). Previous studies have highlighted the association between DNA hypomethylation and overexpression of several genes involved in endometriosis (Meyer et al., 2014; Zidan et al., 2015; Annisa et al., 2018). The following sections will delve into insights of genes associated with endometriosis.

##### 2.1.1.1 Steroidogenic factor (SF-1)

SF-1, encoded by NR5A1 gene, is an orphan nuclear receptor that is implicated in adrenal and gonadal development, steroidogenesis, and reproduction (Luppino et al., 2024). SF-1 is a key regulator of genes involved in cholesterol metabolism, the main source for steroids biosynthesis, namely, Steroidogenic Regulatory Protein (StAR), and CYP19A1 (aromatase) (Luppino et al., 2024). StAR and aromatase are essential for the production of estrogen, following consecutive enzymatic conversions (Zhao et al., 2016). One of the limiting steps in estrogen biosynthesis is the transport of cholesterol into mitochondria, regulated by StAR and aromatase, leading to the conversion of androstenedione to estrogen (Zhao et al., 2016).

**FIGURE 2:**
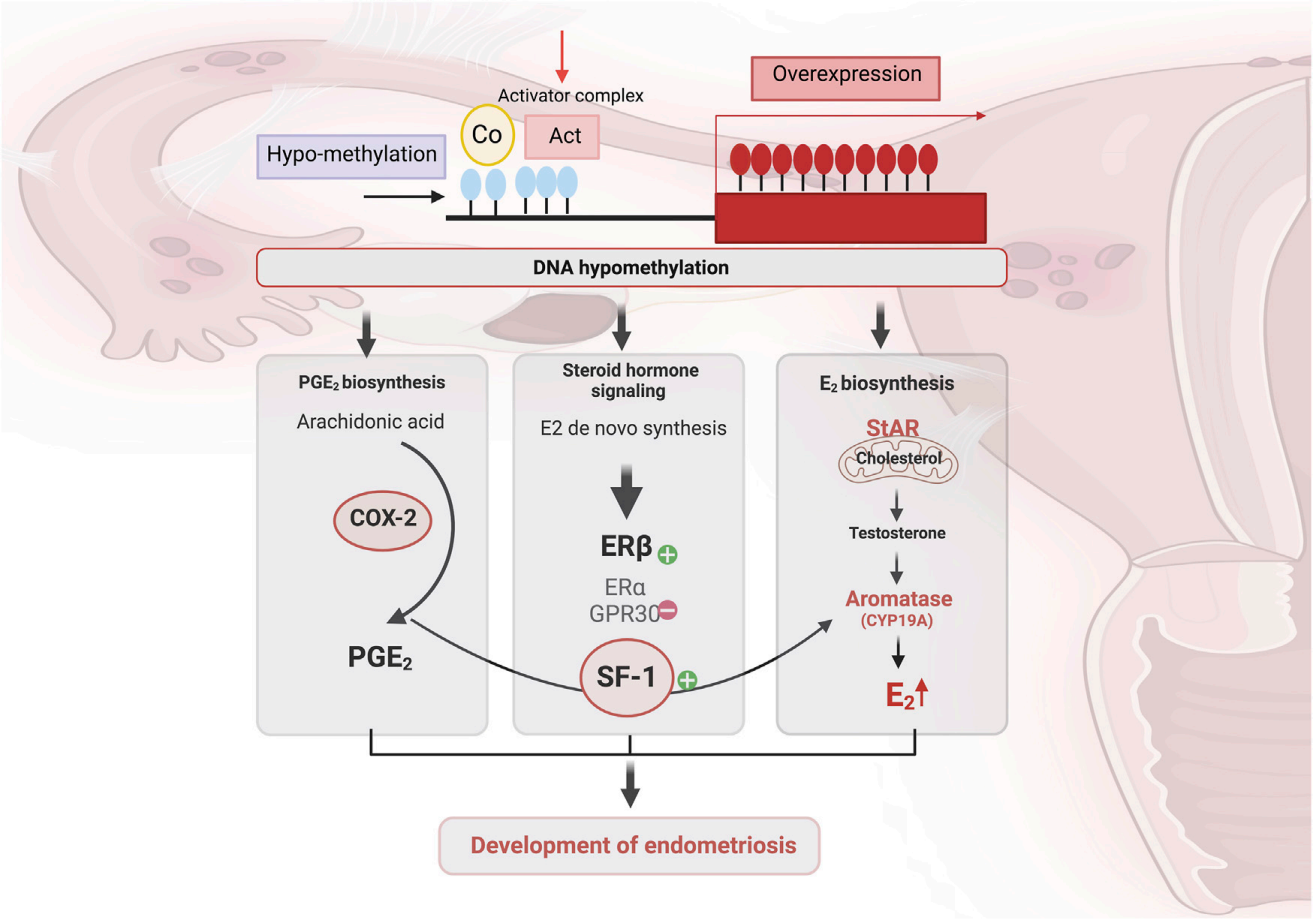
The contribution of DNA methylation in inflammation and the acquisition of steroidogenic capacity and enhances estradiol (E2) signaling in endometriotic women. Global DNA hypomethylation contributes to the upregulation of genes involved in prostaglandin E 2 (PGE 2) biosynthesis and E 2 biosynthesis and signaling. Cyclooxygenase-2 (COX-2) is crucial enzyme in the conversion of arachidonic acid to prostaglandin (PG). Activation of estrogen receptor β (ERβ) increases COX-2 expression, which enhances PGE-2 expression, increasing SF-1, leading to a further increase in estrogen production. The ability to synthesize E2 *de novo* from cholesterol, due to higher expression of steroidogenic acute regulatory protein (StAR) and CYP19A (aromatase), results in local accumulation of E2 in lesions. Created with BioRender (https://www.biorender.com/).

In endometriotic stromal cells, SF-1 directly regulates StAR and aromatase expression (Bulun et al., 2009; Xue et al., 2014). It acts by binding to and activating the promoters of steroidogenic genes, namely, StAR, side-chain cleavage enzyme (SCC), 3-beta-hydroxysteroid dehydrogenase type 2 (HSD3B2), 17-hydroxylase/17,20-lyase (CYP17A1) and CYP19A1. CYP19A1 (Figure 2) (Noël et al., 2010; Bulun et al., 2015). As shown by previous studies, SF-1 is overexpressed in stromal cells from endometriotic tissues compared to eutopic endometrial tissues, contributing substantially to endometriosis (Bulun et al., 2005; Borghese et al., 2008; Utsunomiya et al., 2008; Attar et al., 2009).

Epigenetic silencing of SF1 is lost in endometriosis due to hypomethylation of NR5A1. *De novo* SF1 activation enhances steroidogenic enzyme expression and contributes to the survival of endometrial tissue in ectopic sites, contributing to a hyperestrogenic state and favoring inflammation (Vasquez et al., 2016). The action of estrogen in the endometrium is predominantly mediated by the estrogen receptor α, which is encoded by the ESR1 gene (Bulun et al., 2005). Annisa and colleagues’ study demonstrates a statistically significant difference on methylation profiles of SF-1 in peritoneal endometriosis compared to control groups, as well as between peritoneal and ovarian endometriosis (Annisa et al., 2018). Intriguingly, there is no significant difference of SF-1 promoter methylation between the ovarian endometriosis and control groups (Annisa et al., 2018). In the same line, *de novo* SF1 activation, *in vivo*, promotes aberrant endometrial glands morphogenesis, leading to endometrial architecture disrupting and infertility (Table 1) (Vasquez et al., 2016).

**TABLE 1:** Summary of studies related to aberrantly methylated genes in endometriosis.

Genes	Main findings in the study	References
Steroidogenic factor 1 (SF-1)	Methylation profile of SF-1 promoter did not change significantly in ovarian endometriosis and controls	Annisa et al. (2018)
	SF-1 high expression leads to a lack of uterine decidual response and infertility	Vasquez et al. (2016)
GATA6	GATA6 alone is essential but not sufficient to develop endometriosis. However, the cooperation of GATA6 and NR5A1 is necessary and sufficient for estradiol synthesis, which is essential for the development and persistence of endometriosis	Bernardi et al. (2019)
Cyclo- oxygenase 2 (COX-2)	Hypomethylation of the NF-IL6 site in the COX-2 gene promoter might underline the high expression of COX-2 in both eutopic and ectopic tissues in endometriosis	Zidan et al. (2015)
	Hypomethylation of COX-2 promoter could be responsible for its elevated expression in eutopic endometrium	Wang et al. (2012b)
Estrogen receptor 2 (ESR-2)	ESR2 methylation status was low in both eutopic endometrium and ovarian endometrioma	Maekawa et al. (2019)
	No differences in ESR2 methylation pattern were observed across all cases of intestinal deep endometriosis	Meyer et al. (2014)
Homeobox A10 (HOXA 10)	During the secretory phase, Low expression of HOXA 10 gene was identified along with the hypermethylation as well as higher incorporation of MeCP2 on HOXA 10 promoter in eutopic tissues of women with endometriosis	Samadieh et al. (2019)
	High methylation level was reported in eutopic endometrium of women with endometriosis associated infertility	Muharam et al. (2016)
Progesterone receptor (PR- B)	High methylation at PR-B promoter may be associated with gene expression downregulation, potentially impairing endometrial receptivity in women with endometriosis	Rocha-Junior et al. (2019)
	Compared to normal endometrium (P = 0.000), methylation levels of the PR-B gene promoters showed a significant difference in ectopic peritoneal endometrial tissue (72.40% methylated), ovarian tissue (85% methylated), and eutopic endometrial tissue (72.21% methylated)	Darmawi et al. (2018)
E-cadherin	Compared to normal endometrial tissue, the level of E− cadherin in endometriosis lesions was shown to be lower	Biyik et al. (2021)
	Reduced expression of E-cadherin in the endometrium might be caused by aberrant methylation of the CDH1 promoter region, and this may be linked to the development of ovarian endometriosis in Northern Chinese women	Li et al. (2017)

##### 2.1.1.2 GATA-binding factor 6 (GATA6)

GATA6 is a member of the highly conserved GATA family of transcription factors, which consists of six zinc-finger proteins that regulate stem cell activity and tissue growth (Tremblay and Viger, 2003; Shu et al., 2015). GATA1, 2 and 3 define cell lineage fate during hematopoiesis, while GATA 4, 5 and 6 dictates cell fate in endodermal and mesodermal tissues, including the gonads (Molkentin, 2000). GATA4 and GATA6 are generally expressed in steroidogenic tissues, and are crucial for steroidogenic gene regulation (Tremblay and Viger, 2003; Convissar et al., 2015). Compared to normal endometrial stromal cells, endometriotic tissue exhibit higher levels of GATA6 (Dyson et al., 2014). In ectopic endometrial stromal cells, the overexpression of GATA6 resulting from its hypomethylation, limits the ability to decidualize and has been associated with the transformation of endometrial stromal cells into estrogen-producing endometriosis-like cells (Bernardi et al., 2019). According to a recent study, GATA6 plays an essential role in the acquisition of endometriosis phenotype by endometrial stromal cell (ESC) (Bernardi et al., 2019). However, the acquisition of this phenotype is not sufficient to transform normal endometrial stromal cells, NoEM, into endometriotic-like stromal cells in terms of *de novo* estrogen synthesis (Bernardi et al., 2019). However, the co-expression of GATA6 and SF-1, a key factor in steroidogenesis regulation, is necessary and sufficient to enhance estradiol production by endometriotic cells, a key hormone for the growth and persistence of endometriotic tissue (Table 1) (Lala et al., 1992; Xue et al., 2007; Schimmer and White, 2010).

##### 2.1.1.3 Cyclo-oxygenase 2 (COX-2)

**FIGURE 3:**
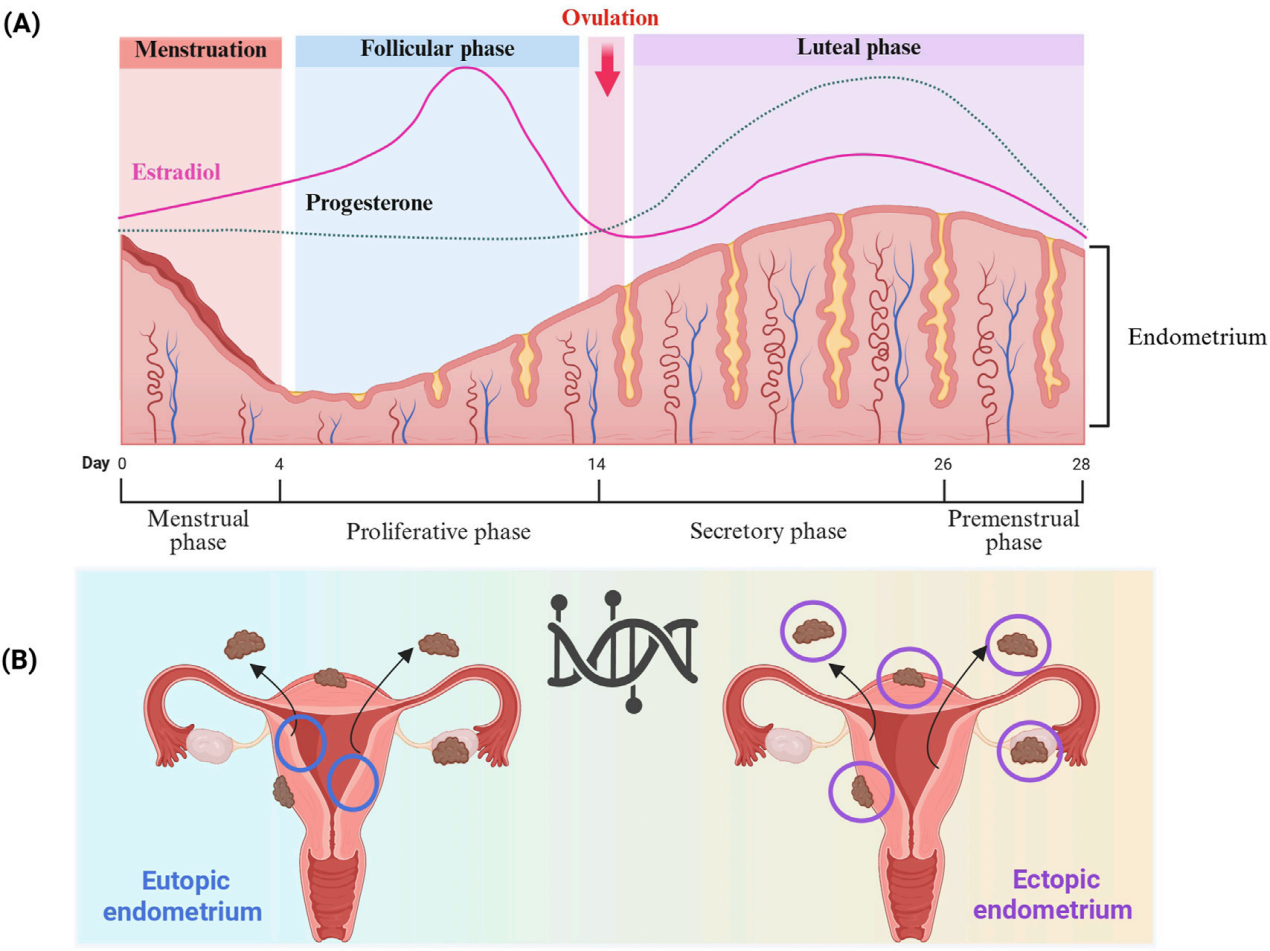
**(A)** Endometrium modulation by steroid hormones across the menstrual cycle. The endometrium undergoes changes during the menstrual cycle, and consists of three main phases: menstrual, proliferative, and secretory, mainly regulated by estradiol and progesterone. During the menstrual phase, estrogen and progesterone levels are at their lowest due to the degeneration of the corpus luteum, which induces the shedding of the endometrium’s functional layer. In the proliferative phase, increasing estradiol levels promote regeneration of the endometrial lining, stimulating epithelial cell proliferation, gland elongation, and revascularization. After ovulation, the corpus luteum produces progesterone drives the onset of the secretory phase. Progesterone drives the endometrial glands to undergo secretory changes and initiates decidualization, which refers to the process by which stromal cells differentiate into specialized decidual cells in preparation for potential embryo implantation. This transformation is vital for establishing a receptive and immunologically supportive microenvironment. In the absence of implantation, reduced levels of progesterone and estradiol trigger endometrial breakdown, leading to menstruation and the start of a new cycle. **(B)** The differences between eutopic and ectopic endometrium in endometriosis, as well as their locations, affect critical biological pathways and contribute to the intra- and inter-lesions heterogeneity. Created with BioRender (https://www.biorender.com/).

**FIGURE 4:**
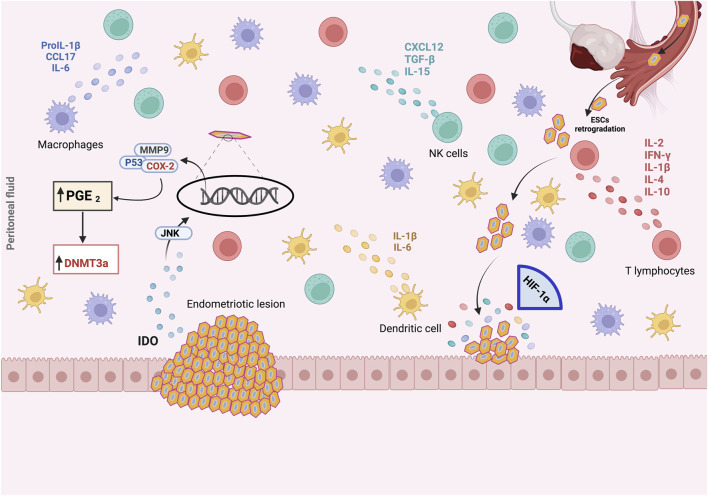
The pro-endometriotic niche and its contribution in the establishment of an advanced lesion. The expression of p53, matrix metalloprotease 9 (MMP9) and cyclo-oxygenase-2 (COX-2) is regulated by the high expression of indoleamine 2,3-dioxygenase (IDO1) via c-Jun N-terminal kinase (JNK) signaling, which enhances the survival of endometriotic stromal cells (ESCs) and inhibits their apoptosis. During the initial ESCs communication with the peritoneal wall, ESCs must face hypoxic stress. As a result, overexpression of hypoxia-inducible factor 1α (HIF-1α) is induced, leading to the secretion of several components into the endometriotic milieu, namely, overexpression of COX- 2, which promotes abnormal production of prostaglandin E2 (PGE2). In the microenvironment, PGE2 is involved in steroidogenesis, angiogenesis and immunosuppression. The recruitment and activation of immune cells, macrophages, natural killer (NK) cells, T lymphocytes, and the secretion of chemokines and cytokines from an existing endometriotic lesion, stimulate the maturation of this immunosuppressive environment and the establishment of advanced endometriosis from the initial lesion, leading to the progression of endometriosis. Created with BioRender (https://www.biorender.com/).

Cyclo-oxygenase (COX) is an enzyme implicated in many physiological and pathological processes (Faki and Er, 2021). Three COX isoforms are known, COX-1, COX-2, and COX-3 (Tyagi et al., 2020). COX-1 and COX-2 are the most studied due to their involvement in both physiological and pathological processes (Rouzer and Marnett, 2009). COX-2 iso-enzyme is usually produced in minimal amounts under normal conditions, but its expression can increase significantly in response to pathological conditions (Pu et al., 2021). Expressed in the glandular epithelium of the endometrium in healthy women, COX-2 expression pattern varies during endometrial cycle phases, notably the proliferative phase and the secretory phase (Figure 3) (Lai et al., 2019). COX-2 expression is lowest at the beginning of the proliferative phrase (Lai et al., 2019). Thereafter, it progressively increases and remains at a high level throughout the secretory phase (Lai et al., 2019). In women with endometriosis, COX-2 expression was significantly increased in eutopic endometrium during the proliferative phase, and in ovarian endometriotic tissue during the secretory phase compared with the control groups (Lai et al., 2019). In addition, women with endometriosis suffering from chronic stress had high COX-2 expression in ectopic lesions (Cho et al., 2010). In the eutopic endometrium, elevated COX-2 expression has been shown to be a result of hypomethylation of the Nuclear Factor site responsible for the Interleukin-6 (NF-IL6) expression site within COX-2 promoter (Zidan et al., 2015). High COX-2 expression leads to Prostaglandin E2 (PGE2) production, and was associated with cell proliferation, migration, invasion, angiogenesis and immunomodulation. Furthermore, such pathway induces expression and enhances activity of aromatase, leading to higher estradiol production (Banu et al., 2008) (Table 1). COX-2 drives pro-endometriotic niche establishment, a favorable and receptive microenvironment undergoing a series of changes during the proliferation of Endometrial Stromal Cells (ESCs), and progression of endometriotic lesions (Burns et al., 2018). The regulation of COX-2 depends on several factors such as Indoleamine 2, 3-dioxygenase (IDO1), through the phosphorylation of the c-Jun N-terminal kinase pathways (Mei et al., 2013). This mechanism enhances ESC survival and inhibits cell apoptosis in the peritoneal cavity. IDO may also regulate immune cell polarization and induce immune tolerance by releasing Interleukin-10 (IL-10) and Transforming Growth Factor-beta (TGF-β) (Figure 4) (Mei et al., 2013; Burns et al., 2018).

##### 2.1.1.4 Estrogen receptor 2 (ER-2)

It is well known that various risk factors, namely, endocrine, genetic, biochemical, environmental and immunological, are involved in the onset and progression of endometriosis (Terzic et al., 2021). In endometriosis, estrogen continues to be the primary trophic element and plays a critical role in the progression of endometriotic lesions (Chantalat et al., 2020). Estrogen acts through at least two ERs subtypes, ERα and ERβ, encoded by the *Estrogen Receptor 1 (ESR1) and 2 (ESR2)* respectively (Song et al., 2022). In target cells, ERs subtypes work both as transcription factors and plasma membrane receptors. Upon Estrogen binding resulting in conformational changes, ERs dimerize, translocate to the nucleus where they interact with estrogen response elements or other transcription factors and engage coactivators to modulate transcription of target genes (Chen et al., 2020; Miziak et al., 2023). Several studies have shown that ESR2 mRNA levels are higher in endometriosis compared to normal endometrium (Lu et al., 2024; Ochoa Bernal and Fazleabas, 2024). Mechanisms behind this overexpression are still unknown (Hu et al., 2022). However, the ERβ promotor region’s hypomethylation may be linked to the overexpression of ERβ in endometriotic tissues (Han et al., 2019; Nazarenko et al., 2019) (Table1).

#### 2.1.2 Hypermethylation

DNA hypermethylation refers to abnormal increases in DNA methylation (Ehrlich, 2019). It can occur in gene bodies and in cis regulatory elements, namely, promoters and enhancers (Ehrlich, 2019). Although studies focused on the tissue specific promoter hypermethylation, at CpG rich promoter regions, tissue specific DNA hypermethylation is more frequently observed within the transcribed gene bodies and in intragenic or intergenic enhancers than in promoters (Ehrlich, 2019). DNA hypermethylation is widely reported as a biomarker across a broad spectrum of diseases, mainly cancer (Zeng et al., 2022; Draškovič and Hauptman, 2024; Li et al., 2025), cardiovascular diseases (Boovarahan et al., 2022), and endometriosis (Darmawi et al., 2018; Elias et al., 2023; Setiawan et al., 2023; Bedrick et al., 2024). The next sections will explore the impact of relevant hypermethylated genes associated with endometriosis.

##### 2.1.2.1 Homeobox A10 (HOXA 10)

Homeobox A10 (HOXA10) is a transcription factor associated with apoptosis and cell proliferation in many types of cancers (Song et al., 2019; Zhang Y. et al., 2019; Jiang and Yang, 2022). In the endometrium, HOXA10 is highly expressed in endometrial glandular and stromal cells under the regulation of several factors such as steroid hormones (Elias et al., 2023). It has been demonstrated that hypermethylation of HOXA10 plays a crucial role in endometriosis and implantation failure in women undergoing *in vitro* fertilization treatment (Taylor et al., 1998; Nazarenko et al., 2019; Samadieh et al., 2019). Several studies have revealed that the level of HOXA10 methylation is significantly higher in the endometrial tissue of women with endometriosis (Elias et al., 2023). They also showed that HOXA10 methylation levels varied according to the type of sample, eutopic or ectopic endometrium, and menstrual cyclicity, proliferative or secretory phase (Elias et al., 2023). They revealed that the level of HOXA10 methylation is considerably higher in eutopic endometrium collected during the secretory phase in patients with endometriosis (Elias et al., 2023). The level of DNA methylation and subsequently HOXA10 expression varies between menstrual cycles, and is coordinated by changes in steroid sex hormone levels (Figure 3) (Yu et al., 2024). In control group, HOXA10 expression is low during the proliferative phase, and increased during the secretory phase, in association with cell differentiation and fibroblast-like endometrial stromal cells conversion into decidual cells, preparing the endometrium for embryonic implantation (Wang W. et al., 2012). Whereas in endometriosis patients, HOXA10 methylation levels increase during the secretory phase, resulting in low HOXA10 expression levels, and thereby cell differentiation inhibition in the eutopic endometrium (Elias et al., 2023). Furthermore, the level of HOXA10 expression is dependent on the methylated site (Elias et al., 2023). It is known that DNA methylation occurring at the promoter region is generally associated with reduced expression (Lee et al., 2020). For HOXA10 gene, studies have shown hypermethylation at the promoter region and at a part of the first exon, the CpG island between −245 bp and 29 bp of the transcription start site (Elias et al., 2023). Furthermore, it has been shown that HOXA10 methylation occurs also in the first and second introns in endometriotic tissues (Elias et al., 2023). This sheds light on the intricate hypermethylation profile of HOXA10 gene, negatively regulating HOXA10 expression and contributing to the heterogeneity of endometriosis (Table 1) (Ji et al., 2017; Samadieh et al., 2019; Elias et al., 2023; Ekanayake et al., 2022).

##### 2.1.2.2 Progesterone receptor b (PR-B)

Progesterone, a steroid hormone synthesized by ovaries, adrenal cortex, and placenta, plays a pivotal roles in female reproductive health and fertility (Zhang and Wang, 2023). It has anti-estrogenic effects, suppresses endometrial proliferation and decidualization, and inhibits the transition of endometrium from the proliferative to the secretory phase (Zhang and Wang, 2023). Furthermore, Progesterone controls embryo implantation, pregnancy maintenance, uterine growth and mammary gland development (Li et al., 2021). Progesterone Receptor B (PR- B) and PR-A represent the two principal isoforms of Progesterone receptors (PGR), transcribed from two promoters of the same gene, and sharing significant overlap in their structural and functional domains (Li et al., 2021). Progesterone, upon binding to its receptor, exerts its effects through the classical pathway inducing conformational changes in the receptor localized in the cytoplasm and translocation to the nucleus, where it initiates transcription of target genes (Mani and Oyola, 2012). Aberrant DNA methylation of PR’s promoter and first exon can mute it at the transcriptional level (Zhang and Wang, 2023). PR-B is a 114 kDa protein with high ligand-induced transcriptional activity (Bedaiwy et al., 2015; Yilmaz and Bulun, 2019). Prior investigations have revealed that in endometriosis, the hypermethylation of the PR-B promoter in ectopic endometrium leads to the suppression of its expression (Yilmaz and Bulun, 2019). Furthermore, the PR-B gene promotor shows elevated methylation exclusively in ectopic endometrial cells (Table1) (Wu et al., 2006).

##### 2.1.2.3 E-cadherin

*CDH1* gene encodes a classical cadherin, E-cadherin, a transmembrane glycoprotein involved in maintaining epithelial cell-cell adhesion (Lialios and Alimperti, 2025). E-cadherin controls multiple processes, namely, cell polarization, migration and cancer metastasis (Zhou et al., 2024). Reduced expression of E-cadherin is a key contributor to the pathogenesis of endometriosis (Matsuzaki and Darcha, 2012; Li et al., 2017; Biyik et al., 2021). It has been reported that the hypermethylation of the CpG island of *CDH1*may contribute to the transcriptional inactivation of the gene (Li et al., 2017). The study conducted by Li and colleagues has reported the CDH1 promoter methylation in eutopic and ectopic endometrium of women with ovarian endometriosis in 26% and 32% respectively, compared to 8% in the endometrial tissue of women without endometriosis (Li et al., 2017). Another research group has shown reduced expression of T-cadherin, E-cadherin, and PR in deep infiltrating endometriosis, with positive correlation among the three markers (Biyik et al., 2021) (Table1).

### 2.2 Histone modification

Histones are proteins that play a crucial role in DNA compaction (Zhang et al., 2021). These proteins support the formation of DNA-protein complex, around 2 m of DNA is packed inside the nucleus (Janna et al., 2020). The nucleosome is the fundamental unit of chromatin, consisting of 4 central histones H2A, H2B, H3 and H4 (Janna et al., 2020). The H1 connects the nucleosomes to form a chromosome (Pathak et al., 2018). Histones have protruding tails that undergo post-translational modifications, namely, acetylation, phosphorylation, methylation, ubiquitylation and sumoylation (Bannister and Kouzarides, 2011; Keck and Pemberton, 2012). Most of these modifications are reversible, making it possible to develop new histone-targeting therapeutic strategies (Lu et al., 2025). It is well established that gene expression and chromatin remodeling depend on DNA methylation and histone modifications (Gagnidze and Pfaff, 2021). However, the mechanisms underlying histone modifications are still not completely understood (Gagnidze and Pfaff, 2021). The most widely recognized histone modifications are acetylation and methylation (Bannister and Kouzarides, 2011; Nasu et al., 2011; Bulun et al., 2019). In endometriosis, current findings have demonstrated the involvement of histone modifications in the pathogenesis of the disease, even if the mechanism is not fully understood (Psilopatis et al., 2023; Bedrick et al., 2024). Moreover, the impact of histone modifications on infertility related to endometriosis is still a subject of research (Psilopatis et al., 2023).

Histones acetylation is one of the first modifications revealed by Allfrey et al. (1964). It involves adding acetyl groups to the N-terminal tails of amino acids such as lysine and arginine, as well as serine, threonine and tyrosine in H3 and H4 molecules (Allfrey et al., 1964). It is regulated by 2 enzymes; Histone Acetyl Transferase (HAT) and Histone Deacetylase (HDAC) (Yang and Seto, 2007). These enzymes influence the binding of histones to DNA, resulting in condensation or decondensation of chromatin, and subsequently gene expression modulation (Bannister and Kouzarides, 2011). In patients with endometriosis, the levels of HDAC1 and HDAC2, the most abundant HDACs in human cells, were found deregulated in endometriotic stromal cells (Hsiao et al., 2017). Several studies have shown that in endometriotic stromal cells, HDAC1 and HDAC2 are upregulated (Colón-Díaz et al., 2012; Samartzis et al., 2013; Xiaomeng et al., 2013). In early proliferative phase, histone acetylation levels are globally increased and progressively decrease during the late proliferative phase until ovulation (Munro et al., 2010). Several studies have shown that overall histone acetylation profiles, particularly H3 and H4 are hypoacetylated in endometriotic stromal cells compared with normal endometrium (Xiaomeng et al., 2013; Monteiro et al., 2014). Furthermore, increased HDAC activity in endometriotic cells leaves promoter regions hypoacetylated, resulting in cell cycle induction and proliferation (Koike et al., 2015). In endometrial epithelial cells, Estradiol and Progesterone significantly downregulated HDAC1 expression (Colón-Díaz et al., 2012). However, in endometrial stromal cells, HDAC2 expression levels were upregulated by Estradiol and downregulated by Estradiol plus Progesterone treatment (Colón-Díaz et al., 2012; Hsiao et al., 2017). This pattern of HDAC1/2 hormonal regulation is lost in the endometriotic cell line, which can be explained by progesterone resistance due to an overall reduction in progesterone receptor levels in endometriotic stromal cells (Bulun et al., 2010).

### 2.3 Non-coding RNAs

Among the crucial components of epigenetic regulation are non-coding RNAs (Liao et al., 2025). They are essential in fundamental biological processes, namely, transcription, genome imprinting, and chromatin remodeling (Liao et al., 2025). They have the particularity of not undergoing the translation process of protein synthesis (Liao et al., 2025). Non coding RNAs can be classified into two categories according to their size, structure, and regulatory properties. Hence, small RNAs refer to RNAs under 200 nucleotides, and long RNAs with more than 200 nucleotides (Chen and Kim, 2024). Over the last two decades, several types of small non-coding RNAs, such as MicroRNAs (miRNAs), PIWI-interacting RNAs (piRNAs), endogenous small interfering RNA (siRNAs), and Small nucleolar RNAs (snoRNAs) have been identified through genetic mapping (Huang Z.hao et al., 2022; Chen and Kim, 2024). With the development of deep sequencing technologies, a new world of small RNA has emerged (Brosnan and Voinnet, 2009). MiRNAs and piRNAs play an pivotal role in germline and somatic cells, respectively through RNA silencing and transposon activity reduction (Saini et al., 2007; Weick and Miska, 2014). On the other hand, long non-coding RNAs modulate the transcriptional and post-translational levels of gene expression (Mattick et al., 2023).

These modulatory RNAs are core elements of cellular machinery that function at several levels to control cellular fate (Yao et al., 2019). In endometriosis, several experiments demonstrated that non-coding RNAs contribute to the pathogenesis of endometriosis (Tables 2–5).

**TABLE 2:** Summary of recent studies evaluating altered circulating miRNAs expression in women with endometriosis.

miRNAs	Sample type	Method	Main findings in the study	References
mir-135a	Plasma	FireFly custom multiplex circulating miRNA assay	A higher level of miR-135a have been noted in women with endometriosis. This result is reliant to the menstrual cycle phase (positive regulation only in the secretory phase) rather than the disease stage	Perricos et al. (2022)
miR-124-3p, miR-6509-5p, miR-548l, miR-26a-2-3p, miR-3622a-3p, miR-3168, miR-29b-1-5p, miR-30e-3p, miR-3124-5p, miR-4511.	Plasma	Artificial Intelligence Machine Learning	Out of the 86 miRNAs included in the ENDO-miRNA study, 10 showed the most potential value, and only miR-124-3p has been cited previously in the context of endometriosis	Bendifallah et al. (2022a)
miR-92b-5p, miR-486-5p, miR-3184-3p, miR-4732-5p, miR-4235p.	Plasma	NGS	A significant upregulation of these exosomal miRNAs has been noted in patients with serous ovarian cancer, extragenital endometriosis and ovarian endometriosis cysts	Iurova et al. (2022)
miR-148a	Serum	qRT- PCR	The levels of miR-148a were considerably lower compared to controls. MiR- 148a promotes apoptosis in endometriosis by targeting the ADAMTS5 gene	He et al. (2022)
miR-26b-5p miR-215-5p miR-6795-3p	Serum	qRT-PCR	These 3 miRNAs are differentially expressed between endometriosis patients and controls. Moreover, they are correlated with disease severity and symptoms such as pain and infertility	Wu et al. (2022)
miR-146a rs2910164 miR-149 rs2292832 miR-196a-2 rs11614913 miR-499 rs3746444	Serum	PCR	The 3 miRNAs variants are suggested to be linked to endometriosis	Farsimadan et al. (2021)
miR-34a-5p miR-200c	Serum	qRT-PCR	Patients with endometriosis had higher level of miR-200c and lower level of miR-34a-5p. The sensitivity of serum miR-34a- 5p and miR-200c was 78.95 % and 100 % ,and specificity was 49.12 % and 100 %, respectively	Misir et al. (2021)
let-7b mir-9	Serum	RT-qPCR	Out of all biomarkers examined, let-7 had the best sensitivity, specificity, and predictive value. Moreover, it was more specific than the cancer antigen CA-125	Pokrovenko et al. (2021)
miR-199a-3p miR-143-3p miR-340-5p let-7b-5p miR-21-5p miR-17-5p miR-20a-5p miR -103a-3p	Plasma	NGS qRT-PCR	Compared to control subjects, patients demonstrated significantly decreased levels of these 8 miRNAs. The range of individual miRNAs' sensitivity and specificity was 0.36 to 1.00 and 0.43 to 1.00, respectively. However, the combination of the five miRNAs (miR-17-5p, miR- 20a-5p, miR-199a-3p, miR- 143-3p and let-7b-5p) produced a sensitivity and specificity of 0.96 and 0.79	Papari et al. (2020)
let-7a-5p let-7b-5p let-7d-5p let-7f-5p let-7g-5p let-7i-5p miR-199a3p miR-320a miR-320b miR-320c miR-320d miR-328-3p miR-331-3p miR320e	Plasma	Microarray	Patients with ovarian endometriosis had considerably lower levels of all 14 miRNAs	Gu et al. (2020)
miRNA-185-5p	Plasma	miRNA sequencing RT-qPCR	MiR-185-5p is a particular biomarker that controls the pathophysiology of endometriosis supporting the notion that treatments targeting miR-185-5p should be prioritized over those that target PDGF and VEGF	Razi et al. (2020)
miR-125b-5p miR-28-5p miR-29a3p	Plasma	small RNA sequencing qRT-PCR	Only miR-125b-5p, miR-28-5p, and miR-29a3p out of the 42 miRNAs identified in the study demonstrated higher diagnostic value (AUC = 60%), with reasonable sensitivity (78%) but poor specificity (37%)	Vanhie et al. (2019)

**TABLE 3:** Summary of recent studies evaluating altered miRNAs expression in tissue of women with endometriosis.

miRNAs	Sample type	Method	Main findings in study	References
miR-21	Normal endometrium Ectopic endometrium Eutopic endometrium	qRT-PCR	MiR-21 is a potential inhibitor of the TGF-β1-SMAD3-ILK signaling pathway that play an essential role in the epithelial mesenchymal transition process	Zubrzycka et al. (2023)
miR-424-5p	Ectopic endometrium Endometrial stromal cells	RT-qPCR	MiR- 424-5p expression was negatively modulated by Circ_0007299 in ectopic endometrial stromal cells	(Mao et al. (2023)
miR-124-3p	Endometrial stromal cells and primary normal endometrial stromal cells from rat models.	RT-qPCR	The inhibition of miR-124-3p expression in ectopic endometrial stromal cells promotes ANTXR2 expression through PCGEM1, involved in endometrial stromal cell proliferation and migration	Liu et al. (2023)
miR-30a-5p, miR-7-5p, miR-143-3p, miR-93-5p	Ectopic and eutopic endometrium of Superficial, deep and ovarian endometriosis Normal endometrium	qRT-PCR	The expression of miR- 93-5p and miR-7-5p was significantly lower in patients with superficial peritoneal endometriosis compared with patients with deep infiltrating and ovarian endometriosis	Antonio et al. (2023)
miR-519b- 3p	Normal endometrium Ectopic endometrium Eutopic endometrium	qRT-PCR	LncRNA HOTAIR controls the miR-519b-3p/PRRG4 pathway, which regulates cell invasion and migration in endometriosis	Bao et al. (2022)
miR-15a-5p	Primary endometrial stromal cells from ectopic, eutopic and normal endometrium	RNA sequencing qRT-PCR	MiRNA-15a-5p was one of the RNA biomarkers related to endometriosis	Wu et al. (2021)
miR-205-5p	Normal endometrium Ectopic endometrium Eutopic endometrium	RT-qPCR	MiR-205-5p was demonstrated to be downregulated in ectopic endometrial tissue compared to eutopic endometrium	Wang et al. (2021b)
miR-9-5p	Ectopic endometrium Eutopic endometrium	qRT-PCR	LINC01116 promotes the development of endometriosis through the miR-9-5p/FOXP1 pathway	Cui et al. (2021)
miR-191, Mir-10b, miR-200c	Eutopic endometrium from women with and without adenomyosis	RT-qPCR	MiR- 10b, miR-200c and miR-191 were significantly dysregulated in the eutopic endometrium of Adenomyosis patients	Borisov et al. (2020)
miR-17-5p	Endometrial tissue	qRT-PCR	MicroRNA-17-5p was up- regulated in patients with endometriosis. Its sensitivity and specificity were 90% and 76.5% respectively	Nabiel et al. (2020)
miR-141-5p	Ectopic endometrium Eutopic endometrium	qRT-PCR	The ectopic endometrium exhibits lower level of miR- 141-5p in women with ovarian endometriosis	Zhang et al. (2019b)
miR-135a/b	Ectopic endometrium Eutopic endometrium	RT-qPCR	Both ectopic and eutopic tissues have elevated levels of miR-135a and miR-135b during the secretory phase	Petracco et al. (2019)

**TABLE 4:** Summary of recent studies evaluating altered salivary miRNAs expression in women with endometriosis.

miRNAs	Sample type	Method	Main findings in study	References
miR-6818-5p, miR-498, miR-1910-3p, miR-3119, miR-501-5p	Saliva	NGS	These miRNAs could be used as a signature for endometriosis-related infertility	Dabi et al. (2023)
Hsa-mir-135a	Saliva	FireFly custom multiplex circulating miRNA assay	Regardless of the stage of the disease or menstrual cycle phase, it has been shown that patients had considerably greater levels of hsa-mir-135a expression in their saliva	Perricos et al. (2022)
miR-34c-5p, miR-19b-1-5p, miR-149-5p, miR-378a-3p	Saliva	NGS	PI3K/Akt, PTEN, Wnt/β- catenin, HIF1α/NF κB, and YAP/TAZ/EGFR are the primary signaling pathways interrupted by these miRNAs	Bendifallah et al. (2022b)

**TABLE 5:** Summary of recent studies evaluating altered long RNAs expression in women with endometriosis.

lncRNA	Sample type	Method	Main findings in study	References
SNHG4	Tissue slices embedded in paraffin blocks from women with and without endometriosis	qRT-PCR	SNHG4 expression was found to be higher in patients than the control group	Szaflik et al. (2023)
LINC01960-201	Endometrial stromal cells	RT-qPCR	In women with endometriosis, LINC01960-201 is considered a key regulator of the decidualization of endometrial stromal cells during the implantation window	Cai and Lang (2022)
HOTAIR	Ectopic and eutopic endometrium from patients with ovarian endometriosis Normal endometrium Endometrial stromal cells	qRT-PCR	HOTAIR regulates the invasion and migration capacity of endometrial stromal cells, by modulating the miR-519b-3p/PRRG4 pathway	Bao et al. (2022)
H19	Endometrial stromal cells from ectopic and eutopic endometrium with endometriosis. Endometrial stromal cells from normal endometrium	qRT-PCR	Estrogen controlled the expression and the function of lncRNA-H19 in ectopic endometrial stromal cells	Liu et al. (2022b)
H19	Normal endometrium Ectopic endometrium Eutopic endometrium	Chromatin immunoprecipitation (ChIP) assay qRT-PCR	The expression and epigenetic alterationof H19 probably downregulate IGF1 and IGF2 expression in endometriosis	Kamrani et al. (2022)
ADAMTS9- AS1	Ectopic and eutopic endometrium of patients and murine model Primary murine endometrial stromal cells from eutopic and ectopic tissues.	qRT-PCR	Through the miRNA/GPX4 axis, ADAMTS9- AS1 controls ferroptosis resistance in endometriosis, promoting the proliferation of endometrial stromal cells and controlling miR- 6516-5p/GPX4-dependent ferroptosis	Wan et al. (2022)
UCA1, MALAT1 TC0101441 and H19	Tissue slices embedded in paraffin blocks from women with and without endometriosis	RT-qPCR	H19 was the only lncRNA that demonstrated a significant association with endometriosis On the other hand, UCA1, MALAT1, and TC0101441 did not significantly affect the risk of endometriosis	Szaflik et al. (2022)
LINC02381, IGFL2-AS1	Normal endometrium Ectopic endometrium Eutopic endometrium	RNA-sequencing RT-qPCR	Endometriosis and normal endometrial tissues showed significantly different expressions of IGFL2-AS1 and LINC02381	Yin et al. (2022)
LINC00339	Endometrial tissue Ectopic tissue	RNA-sequencing qRT-PCR *In situ* hybridization	Immune defense pathway gene expression was significantly affected by manipulation of LINC00339 expression in endometrial stromal cell lines	Holdsworth-Carson et al. (2021)
H19, GS1-358P8.4, RP11-96D1.10	Ectopic endometrium Eutopic endometrium	RNA-seq data from the Gene Expression Omnibus (GEO)	LncRNAs H19, GS1-358P8.4 and RP11-96D1.10 are significantly associated with ovarian endometriosis	Bai et al. (2021)

#### 2.3.1 Micro-RNAs

MiRNAs consist of 17–25 nucleotides and represent 1% of the human genome (Friedman et al., 2009). Several experimental studies have shown that miRNAs regulate numerous biological processes and have been implicated in many diseases (Cui et al., 2024). Currently, the miRNAs database miRbase contains 1917 human miRNAs (Kozomara et al., 2019). miRNAs control gene expression through binding to mRNAs, thereby regulating different intracellular pathways (Bartel and Chen, 2004). Experimental analyses and databases such as miRBase have been used to identifie new miRNAs using reference sequences obtained from databases, such as NCBI-BLAST, RNAfold, RNAHybrid and other programs for the identification of miRNAs and their targets (Yao et al., 2019; Altschul et al., 1990; Krüger and Rehmsmeier, 2006; Lorenz et al., 2011). Several methods are employed in experimental practice, including Northern blot which is less frequently used due to the advent of microarrays and qPCR (Siddika and Heinemann, 2021). However, Northern blot is still the reference as it detect precursors miRNAs (pre-miRNA) and mature miRNAs without amplification bias (Siddika and Heinemann, 2021). Microarrays are high-throughput screening system for miRNAs identification and expression analysis, as well as to compare miRNAs expression levels in different tissues and species (Wang et al., 2018a). Reverse Transcription Quantitative PCR (RT-qPCR) is recognized as a technique with low to moderate throughput, appropriate for studying miRNAs levels and functions (Salone and Rederstorff, 2015). Next-Generation Sequencing (NGS) analysis is more used for miRNAs variants identification, not recovered by the conventional targeted methods such as RT-qPCR and microarrays (Willenbrock et al., 2009; Liu et al., 2011). In endometriosis, miRNAs profiling is used to compare miRNAs expression profile between women with and without endometriosis (Papari et al., 2020; Bendifallah et al., 2022a; Brady et al., 2024). It is well established that miRNAs are implicated in several cell signaling pathways involved in endometriosis development (Zhang et al., 2024a). Differential expression of miRNAs has been observed in tissues, body fluids, and saliva (Papari et al., 2020; Bendifallah et al., 2022b; 2022a). The following paragraphs explore circular, tissue and salivary miRNAs.

##### 2.3.1.1 Circulating micro-RNAs

Vanhie et al., conducted a genome-wide miRNAs expression analysis using small RNA sequencing to identify a set of miRNAs differentially expressed between women with and without endometriosis (Vanhie et al., 2019). RT-qPCR was applied to assess the expression of 41 miRNAs, and 3 diagnostic models were developed to differentiate between controls and different endometriosis stages: minimal to mild endometriosis, and moderate to severe endometriosis (Vanhie et al., 2019). For minimal to mild endometriosis, the model involving miR-125b-5p, miR-28-5p and miR-29a-3p had an AUC of 60%, with an acceptable sensitivity of 78%, though its specificity was limited at 37% (Vanhie et al., 2019). Moustafa et al., have shown that women with endometriosis had considerably higher expression levels of 4 serum miRNAs; miR-125b-5p, miR-150-5p, miR-342-3p and miR- 451a (Moustafa et al., 2020). However, two serum miRNAs exhibited notably lower levels in the endometriosis group; miR-3613-5p and let-7b (Moustafa et al., 2020). These miRNAs demonstrate a high ability to identify endometriosis and other gynecological pathologies with an AUC> 0.9 in two independent studies (Moustafa et al., 2020). The ENDO-miRNA study included 86 miRNAs, 10 of them have revealed a greatest potential value; miR-124-3p, miR-6509-5p, miR-548L, miR-26a-2-3p, miR-3622a-3p, miR-3168, miR-29b-1-5p, miR-30e-3p, miR-3124-5p, miR-4511. Among the10 miRNAs identified, only miRNA124-3p has been documented in association with endometriosis (Bendifallah et al., 2022a). Table 2 summarizes recent findings on circulating miRNAs as potential biomarkers for endometriosis diagnosis.

##### 2.3.1.2 Tissular micro-RNAs

Numerous research teams have used microarrays or NGS technologies to identify miRNAs transcripts that are distinctly expressed in ectopic lesions, ovarian, peritoneal, or rectovaginal, compared to paired or unpaired eutopic tissues (Saare et al., 2017). However, there was a lack of agreement between the findings of various studies. Many studies compared whole lesions with endometrial tissue, others compared endometrium from patients and controls, and some used pure isolated cell fractions from lesions and endometrium (Saare et al., 2017). These discrepancies between studies stem from the sample composition. Hence, the heterogeneity of tissue composition could explain the discordant results between the different studies (Table 3) (Saare et al., 2017).

##### 2.3.1.3 Salivary micro-RNAs

Researchers have recently begun the work on salivary miRNAs as a non-invasive diagnostic tool for endometriosis. Table 4 summarizes the studies that have been carried out on miRNAs in saliva.

#### 2.3.2 Long non-coding RNAs

All RNAs with more than 200 nucleotides and low protein encoding potential are referred to long non-coding RNAs (lncRNAs) (Gil and Ulitsky, 2020; Zhang Q. et al., 2020). Accumulating evidence has highlighted the contribution of lncRNAs to several human diseases, namely, cancer, cardiovascular and autoimmune diseases (Zhang Q. et al., 2020; Zhao et al., 2020). Currently, Several research studies have shown that lncRNAs enhance the onset and development of endometriosis (Lin et al., 2019; Liu et al., 2020). In ovarian endometriosis, the first microarray-based research on lncRNA expression has revealed 948 LncRNA and 4,088 mRNAs transcript dysregulation in ectopic endometrial tissue, compared with paired eutopic endometrial tissue (Feng and Tan, 2020). Table 5 shows recent findings on abnormal lncRNA expression in women with endometriosis.

### 2.4 Epigenetic of endometriosis immune microenvironment

The Epigenetic modifications have a significant role in modulating the endometriosis immune microenvironment (Szukiewicz, 2022; Abbaszadeh et al., 2023; Shi et al., 2025). The immune system holds remarkable potential to recognize and eliminate endometrial implants in the peritoneal cavity (Suszczyk et al., 2024). However, in endometriosis, inflammation and altered immune system, including impaired natural killer (NK) and macrophages activity, T-helper1 (Th1)/T-helper2 (Th2) imbalance, and elimination of the regulatory function of T cells, reduce the clearance of regurgitated endometrial cells and elicits the oxidative stress response and inflammation (Szukiewicz, 2022; Abbaszadeh et al., 2023). The dysregulation of Th1/Th2 and Th17/Treg balances were associated with endometriotic lesions progression, through the abnormal cytokine secretion and enhanced inflammation (Le Menn et al., 2022; Szukiewicz, 2022). T cells dysfunction, including impaired cell proliferation, inflammation, immunogenicity of endometriotic stromal cells, angiogenesis, and sex steroid hormone responsiveness, are relevant mechanisms underlying the pathophysiology of endometriosis (Szukiewicz, 2022). The immune landscape-endometriosis crosstalk involves an interplay between T cells, prostaglandins (PGE2), metalloproteinases (MMP-2, -3, -9), cytokines (TNFα, IL-1β, IL-8, IFNγ, MCP-1, and MIF) and adhesive molecules (VCAM-1, ICAM-1) (Figure 4) (Chopyak et al., 2022). Shifting the Th1/Th2 balance to favor the Th2 phenotype is one of the most critical immunological features of endometriosis. Furthermore, accumulating data suggests that Th17 and Treg play a significant role in clearing refluxed endometrial tissue. IL-17a, inflammatory mediator, associated with TNFa, boost the secretion of IL-8 and COX-2 in a p38 MAPK, p42/44 MAPK, and stress-activated c-Jun N-terminal kinase dependent manner (Hirata et al., 2008). Interestingly, debris clearance is more effective when the Th17/Treg balance tips in favor of Th17, associated with IL-6 and IL-17 inducing inflammation (Hirata et al., 2008; Gogacz et al., 2016; Tanaka et al., 2017).

It is worth emphasizing that epigenetic modifications are among the factors modulating the immune landscape of endometriosis (Shi et al., 2025). Epigenetic modifications can directly modulate the immune microenvironment. Abnormal epigenetic regulation is closely associated with the occurrence and development of many diseases, with DNA methylation and post-translational modifications (PTMs) are the most common abnormal epigenetic mechanisms strongly associated with various disorders (Tsankova et al., 2007; Orioli and Dellambra, 2018; Lu et al., 2020). Accumulating evidence has shown the contribution of PTMs including phosphorylation, methylation, acetylation, glycosylation, lipidation, ubiquitination, and SUMOylation in Th1/Th2 and Th17/Treg imbalances through the key molecules involved in their differentiation and function (Le Menn et al., 2022; Riaz et al., 2023). In instance, the major regulatory transcription factors such as RORγt (retinoic acid-related orphan receptor gamma t) and Foxp3 (forkhead box P3) are directly regulated by PTMs (Le Menn et al., 2022; Szukiewicz, 2022; Le Menn et al., 2022; Szukiewicz, 2022). Regarding inflammation, non-coding RNAs play pivotal role in inflammatory responses and during activation of inflammasomes (Abbaszadeh et al., 2023).

Findings have indicated that miRNAs in endometrial tissue play a key role in modulating the expression of inflammatory mediators. It has been reported that miR-199a was linked to the inhibition of paramount regulator of inflammation NF-κB through the downregulation of inhibitor of nuclear factor kappa B (IκBα) (Abbaszadeh et al., 2023). miR-182 has also the potential of inhibiting NF-κB pathway by targeting one of its related transcriptional factors p65, inducing inflammation and promoting the establishment of endometriotic lesions (Abbaszadeh et al., 2023).

Aberrant function of almost all types of immune actors has been reported in endometriosis, including altered T-cell and NK cytotoxicity function, polyclonal B cells activation, enhanced peritoneal macrophages recruitment, and inflammation (Osuga et al., 2011; Ahn et al., 2015; de Barros et al., 2017; Riccio et al., 2018; Agostinis et al., 2021; Szukiewicz, 2022). Epigenetic reprogramming of T cells in endometriosis has now been well recognized. In endometriosis, a significant decrease in cytotoxic T cells frequency associated with impaired function has been demonstrated. In T cells, the altered apoptotic pathways have been suggested to be linked to DNA hypermethylation and chromatin structure changes in the perforin gene regulatory elements (Lu et al., 2003; Szukiewicz, 2022). In the other hand, IL-6, upregulated in endometriotic stromal cells, plays a significant role in Th2 differentiation. Recent findings show the IL-6 pathway regulation by DNA methylation, miRNAs, and posttranslational modifications (Candido et al., 2021; Lamprianidou et al., 2021).

According to Lin et al., endometriotic lesions show higher miR-20a levels, with the potential of enhancing PGE2 production, and thereby contributing to inflammation (Lin et al., 2012; Szukiewicz, 2022). It is noteworthy that PGE2 plays a significant role on immune cell functions, such as macrophages and NK cells (Hsiao et al., 2014; Mei et al., 2018). In endometriosis, Inflammation exacerbation can result from low levels of some miRNAs, such let-7b and miR-215-5p and high levels of some others, such as miR-20a and miR-125-5p, individuals compared to the control group (Abbaszadeh et al., 2023).

The polarization of the macrophages into M2, through PI3K signaling pathway, is another hallmark of endometriotic immune microenvironment. In endometriosis, peritoneal fluid or medium from cultured peritoneal macrophages exhibit higher IL-10 levels (Ramírez-Pavez et al., 2021). It is well established that miR-301a-3p and miR-887-5p promote M2 polarization and IL10 secretion (Suen et al., 2014; Huang et al., 2022a; 2022b).

Zheying Liu et al. have shown that along with reduced lncRNA H19 levels, miR-342-3p show higher serum expression level. Furthermore, miR-342-3p binds to the 3′UTR of Immediate early response gene (IER3) to suppress its expression, ending up with high level of TGF-β and RORγt, a master regulator of the Th17 cell lineage (Liu et al., 2019; Ghafouri-Fard et al., 2020).

Supplementary Material summarizes differential expressed miRNA and LncRNAs having roles in immune system response in endometriosis. Future studies are required to analyze how aberrant epigenetic modifications, notably PTMs can be a potential for failure of immune system in clearing endometriotic cells.

## 3 Discussion

Endometriosis is typically one of the main causes of pelvic pain and infertility, impacting women’s health worldwide (Kirk et al., 2024; Skorupskaite and Bhandari, 2024). Although endometriosis is common, the usual diagnostic delay is 7–10 years, making it a serious public health concern. This delay is mostly caused by the lack of accurate, accessible, and non-invasive diagnostic tools (De Corte et al., 2024). Epigenetic mechanisms play a key role in the endometriosis pathophysiology and hold potential promise as diagnostic biomarkers (Ducreux et al., 2025). In the recruiting clinical trial (NCT06572852), investigators hypothesize that differential methylation profiles, integrated with genetic, epigenetic, and clinical data, can accurately classify endometriosis cases.

### 3.1 Epigenetic biomarkers and diagnostics

Compared to transcriptomic biomarkers, epigenetic biomarkers present several advantages namely, a high stability in multiple biological samples including fluids (plasma, serum, urine, saliva, semen, and vaginal secretion), and tissues (fresh, frozen, and FFPE tissues) (García-Giménez et al., 2017). Epigenetic biomarkers offer information on disease progression, making them valuable as biological fingerprints. Moreover, epigenetic biomarkers can reflect environmental and lifestyle influences (García-Giménez et al., 2017) (Anastasiu et al., 2020) (Toiyama et al., 2014; Taryma-Leśniak et al., 2020).

Currently, a significant body of research is dedicated to DNA methylation and miRNAs (Toiyama et al., 2014). Regarding DNA methylation, studies have reported that the methylome of cancer cells are distinct from healthy cells. Given the tissue-specific DNA methylation patterns, methylome can be used to distinguish between various cancer types (Rendek et al., 2024). Its note emphasizing that the methylome of cancer can be analyzed in different body fluids, mainly blood liquid biopsy (Wang and Valent, 2009). Besides its lower cost and minimally invasive nature, liquid biopsies exhibit the ability to track the progression of malignant tumors, whether primary or metastatic, and recognize the tumor recurrence (García-Saenz et al., 2017; Insua et al., 2017). Furthermore, the DNA methylation Profile is transmitted with high fidelity to daughter cells, making it advantageous for *in vitro* diagnostic tests (Taryma-Leśniak et al., 2020). Furthermore, DNA methylation is preserved despite variations in clinical sample handling procedure and storage (Kristensen et al., 2009). Thus, these features underscore the potential use of DNA methylation as IVD assays in cancer. Currently, available methylation-based liquid biopsy tests are designed for single cancer detection, applied for colorectal cancer, lung cancer, bladder cancer and liver cancer, or multi-cancer detection. Concerning colorectal cancer, almost commercially available tests use stool samples as source of DNA, namely, Colovantage (Warren et al., 2011), CologuardTM (Warren et al., 2011; Onieva-García et al., 2015), and ColoSureTM (Ned et al., 2011). Few blood-based tests are available on the market, limited to Epi proColon 2.0 CE, COLVERA and Nu.Q™ (Rendek et al., 2024). In colorectal cancer, a study performed with 9,989 subjects have demonstrated that Cologuard^®^ has a sensitivity and a specificity for colorectal cancer detection of 92.3% and 86.6%, respectively (Imperiale et al., 2014). The Nu.Q^®^ assay has also demonstrated a sensitivity of 91.2% for Colorectal cancer and 83.0% for high risk adenoma (Herzog et al., 2017).

In lung cancer the available validated epigenetic biomarker tests are EarlyTect^®^ and Epi ProLung. Regarding multiple cancer screening, Galleri^®^ test, PanSeer, IvyGene and CancerRadar are developped (Rendek et al., 2024). More than 30 DNA methylation-based assays to aid clinical decision making in cancer have reached the market, an unequivocal indicator of DNA methylation-based test growing market size (Davalos and Esteller, 2023).

In the other hand, miRNAs stand out as a great candidate for diagnostic applications. miRNAs have the particularity to be protected from degradation by exosomes during migration out of cells and into body fluids (Spada, 2021). Moreover, accessing sequencing data from circulation is relatively simple, making the detection of differentially expressed miRNAs, and correlation with therapeutic response establishment possible (Condrat et al., 2020; Hussen et al., 2021). Liquid biopsy markers include circulating tumor cells, ctDNA, exosomes, free miRNA, lncRNA, circRNA, proteins, and so on (Ma et al., 2024). MiRNAs have been proven to be a good noninvasive cancer biomarkers, namely, due to their enhanced expression levels in patients and ease of detection. Furthermore, expression levels can reflect treatment response and predict prognosis. In blood of cancer patients, exosomal miRNAs are stable and correspond closely to the expression profile in the tumor. It has been shown that material extracted from liquid biopsy often carry higher quality than that from a tissue biopsy. Hence, blood liquid biopsy emerges as a potential diagnosis and prognosis tool, easy to perform, minimally invasive, and can be repeated multiple times (Heidrich et al., 2021; Jung et al., 2021; Bagheri et al., 2024).

Epigenetic biomarker development faces many challenges for clinical application. One of these challenges is to discover potential candidates with rigorous evaluation of their specificity and sensibility in large scale validation trials (Wu et al., 2021). MiRNAs use as biomarkers experience challenges linked to several factors such as, appropriate control groups, sample sizes, sample collection and processing methods, independent validation, post-analysis of candidate biomarkers, and studies of differential miRNA expression in body fluids. The potential confounding effects of background factors needs to be taken into account (Takizawa et al., 2022).

Moreover, the extraction and purification of miRNAs are essential steps to accurately identify miRNAs (García-Giménez et al., 2017). Thus, the standardization of these methods is a critical step for the reproducibility and the replicability of studies. The use of endogenous reference miRNAs in RT-qPCR to normalize Cq values and minimize technical variations is an important step (Faraldi et al., 2019).

Despite the extensive research in epigenetic modifications, especially DNA methylation and miRNAs, only a small number of biomarkers have reached clinical application. This points to a critical need to enhance efforts toward their clinical implementation. Thus, the standardization of pre-analytical techniques, improved assay methodologies, and an enhanced understanding of the biological mechanisms underlying epigenetic patterns efforts are fundamental to resolving current challenges and advancing both foundational and translational research.

### 3.2 Epigenetic of endometriosis

DNA methylation stands as the most frequent epigenetic modification in the endometrium (Adamczyk et al., 2022). The SF-1 gene promoter in endometriosis is specifically hypomethylated in peritoneal endometriosis (Annisa et al., 2018). Meanwhile, in a previous study realized by Noël et al., it has been shown that the SF-1 protein expression was undetectable in all type of endometriosis; peritoneal, ovarian, or deep infiltrating endometriosis (Noël et al., 2011). The GATA6 alone is essential in endometriosis pathogenesis but not sufficient to confer an endometriosis phenotype (Bernardi et al., 2019). In the same line, the cooperation between GATA6 and SF-1 is reported to be sufficient for endometriosis development and persistence (Bernardi et al., 2019). In endometriotic cells, Izawa et al. identified a specific region in GATA6 gene body with hypomethylated CpGs (Izawa et al., 2019).

Concerning hypermethylation, the most studied gene is HOXA10 and several studies have shown the link between its hypermethylation and endometriosis (Ji et al., 2017; Elias et al., 2023). Patients with endometriosis have decreased expression of HOXA10 in the eutopic endometrium during the secretory phase (Samadieh et al., 2019). The genes mentioned in Table 1 (SF-1, GATA6, COX-2, ESR-2, HOXA10 and PR-B) are the most likely to account for endometriosis onset and development. A recent study by Lei and his colleagues, based on NGS profiling, have identified 1,837 differentially expressed genes, including 1,079 upregulated genes and 758 downregulated genes in the ectopic groups (Lei et al., 2023). Additional confirmation of the highest-ranked genes involved in differential methylation revealed that Transmembrane Protein 184A (TMEM184A), Stratifin (SFN), Killer Cell Immunoglobulin Like Receptor three Ig Domains X1 (KIR3DX1), Estrogen Receptor 1 (ESR1), Phosphatidylinositol-4,5-bisphosphate 3-kinase Catalytic subunit Gamma (PIK3CG) and Ribonuclease A family member 1, pancreatic (RNASE1) were relevant candidate genes in ovarian endometriosis (Lei et al., 2023). Furthermore, this study stands out for having established a link between infection with the human papillomavirus (HPV) and endometriosis. The study revealed that hypermethylated and hypomethylated genes in ectopic environments were enriched in HPV infected tissue (Lei et al., 2023).

Histone modifications and their contribution in the endometriosis are still unclear (Psilopatis et al., 2023). This gap in knowledge results of the restricted set of research that have worked on this component. In endometriosis, The most reported histone modifications are acetylation and methylation (Bedrick et al., 2024). However, histone phosphorylation and ubiquitination studies are still lacking. One of the pioneer studies reported in endometriosis histone modifications profiling was conducted in 2013 by Xiaomeng et al. (Xiaomeng et al., 2013). First, they revealed low levels of histone H4 acetylation in eutopic and ectopic endometrial tissues. Furthermore, the ectopic endometrium showed a notable decrease in HDAC1 mRNA levels, while in eutopic endometrial tissue, HDAC2 mRNA expression was significantly increased (Xiaomeng et al., 2013). In 2019, Kim et al. found that infertile women with endometriosis had reduced levels of HDAC3 in their eutopic endometrium (Kim et al., 2019). In 2022, the same research team studied the role of NAD + dependent class III HDAC Sirtuin 1, a stress-response and chromatin-silencing factor, showing notable increase in Sirtuin 1 expression in epithelial and stromal cells from endometriosis patients (Kim et al., 2022). Furthermore, high levels of Sirtuin 1 in endometriosis lesions appeared to cause further aggravation of endometriosis symptoms (Kim et al., 2022). As a final consideration, histone modifications seem to have a greater significance in endometriosis pathogenesis (Psilopatis et al., 2023). However, future studies should improve methodology and investigate the specific mechanisms by which histone modifications influence endometriosis pathogenesis.

Several studies using NGS technologies have recently attempted to identify non-coding RNAs differentially expressed in endometriosis, not only for their potential clinical application as diagnostic or prognostic biomarkers of the disease, but also to better understand the pathogenesis of endometriosis (Hudson et al., 2021). To date, several studies have demonstrated the role of non-coding RNAs in the pathogenesis of endometriosis, especially miRNAs and lncRNAs (Maier and Maier, 2021; Bendifallah et al., 2022a; Liang et al., 2022; Abbaszadeh et al., 2023; Ravaggi et al., 2024; Oghenemaro et al., 2025). lncRNA and miRNA expression profiles have been investigated in various samples, endometrial tissue, blood and saliva, collected from patients with endometriosis (Petracco et al., 2019; Cui et al., 2021; Bendifallah et al., 2022a; 2022b; Cai and Lang, 2022) it is noteworthy that functional interactions exist between these two sets of transcripts, miRNAs and lncRNAs, with a number of miRNAs being inhibited by lncRNAs (Meng et al., 2021; Gao et al., 2025). In instance, it has been demonstrated that the lncRNA H19 acts as a molecular sponge and reduces the availability of let-7 miRNA (Ghazal et al., 2015). This let-7 downregulation increases the proliferation of endometrial stromal cells through Insulin-like Growth Factor 1 Receptor (IGF1R) overexpression (Ghazal et al., 2015). Evaluation of the RNA interaction network in endometriosis has revealed the role of miRNAs and lncRNAs associated with growth and apoptosis genes regulation in endometrial stromal cells, namely, Cyclin-Dependent Kinase 1 (CDK1) and Proliferating Cell Nuclear Antigen (PCNA) (Zhang M. et al., 2020). In the microenvironment level, another research group reported the importance of the H19/miR-342-3p/IER3 pathway in reducing the risk of endometriosis through suppressing Th17 cell differentiation (Liu et al., 2019). In ovarian endometriosis, it has been shown that CDKN2B antisense RNA 1 (CDKN2B-AS1) regulates AKT serine/threonine kinase 3 (AKT3) expression by sponging miR- 424-5p (Wang S. et al., 2021).

### 3.3 Environmental factors

The interplay between environmental factors and epigenetics is increasingly established in endometriosis. Given that endometriosis is an epigenetic disease, the influence of lifestyle factors such as smoking, alcohol, dietary factors, phytoestrogens, physical activity, stress, and infections, remains an area of ongoing investigation, with findings varying depending on study populations and methodologies (Hemmert et al., 2018; Coiplet et al., 2022). It has been demonstrated that perinatal and childhood environmental exposures are positively linked to endometriosis, including intrauterine tobacco exposure, low birth weight, and pet exposure during childhood (Amazouz et al., 2025). A recent umbrella review meta-analysis of 354 observational studies with a population of over 5 million, has provided a detailed review and critical analysis of environmental risk factors associated with endometriosis (Zhang and Ma, 2021). In this study, a total of 40 risk factors, including lifestyle, reproductive factors, early life factors, race and ethnicity, and others were assessed for their association with endometriosis (Zhang and Ma, 2021). Among these factors, only alcohol intake and exposure to endocrine disrupting chemicals showed a strong link to endometriosis (Zhang and Ma, 2021).

In the same line, endocrine disrupting chemicals, namely, benzophenone and paraben families, harmful chemicals often found in cosmetics and personal care products, have been linked to heightened risk of endometriosis (Peinado et al., 2021).

In the current state of the fight against endometriosis in the European Union, the European Parliament emphasized the high-risk association of pollutants, namely, polychlorinated biphenyls, organochlorine pesticides and dioxins with endometriosis (Parliamentary question, 2023). Pointing that exposure to polychlorinated biphenyls is associated with 70% increased risk of developing endometriosis. Similarly, exposure to dioxins raises the risk by 65%, while exposure to organochlorine pesticides is linked to a 23% increase in risk (Parliamentary question, 2023). The complex nature of these chemicals co-existing as mixtures in the environment makes risk evaluation difficult (Bruner-Tran and Osteen, 2010; Yao et al., 2017). Few Epigenetic studies carried out on the interplay between environmental factors and epigenetic modifications, highlighting the need for more well-designed, sufficiently powered studies.

### 3.4 Epigenetic tools and databases

The expanding volume of epigenomic data calls for advanced database that can store, standardize, and facilitate the exploration of epigenomic patterns, namely, DNA methylation, histone modifications, and non-coding RNAs. Among the key databases used in epigenetic research is EpiFactors (http://epifactors.autosome.org), a manually curated database, offering information about epigenetic regulators, their molecular complexes, targets and products (Marakulina et al., 2023). The latest version of EpiFactors includes data on 902 proteins, comprising 101 histones and protamines, along with a newly compiled collection of 124 lncRNAs (Marakulina et al., 2023). Besides EpiFactors, various open-access databases field are available. Regarding DNA methylation, there are several databases that offer data on methylation patterns obtained across normal and pathological conditions, such as methDB (http://www.methdb.net/), NGSmethDB (http://bioinfo2.ugr.es/NGSmethDB), MethBank (https://ngdc.cncb.ac.cn/methbank/), MethHC (http://methhc.mbc.nctu.edu.tw), and The Cancer Genome Atlas (TCGA) (Ghai et al., 2020; Shang et al., 2022; Ragini et al., 2023; Zhang et al., 2023). Nevertheless, a gap remains in detailed knowledge about the proteins involved in establishing or performing active DNA demethylation, especially when linked to their expression in different cell types and conditions (Medvedeva et al., 2015). Concerning histone modifications, database such as Histone Modification Database (HHMD), Histone Database, and HIstome database are used (Medvedeva et al., 2015). About miRNAs, the identification of miRNAs-target interaction (MTI) is crucial for biological processes annotation and therapeutic strategies development (Cui et al., 2023). Numerous databases of miRNAs are available, namely, HMDD (Human MicroRNA Disease Database), which is a continuously updated by the integration of experimentally verified miRNA–disease associations (http://www.cuilab.cn/hmdd). Compiled from biomedical literature, HMDD features 53,530 documented association between 1871 miRNAs and 2,360 distinct diseases (Cui et al., 2023; Cui et al., 2024). Expanded miRNATissueAtlas2, is a database that compiles miRNAs expression atlas based on 46,997 human tissue samples from 74 different organs, including physiological tissues, cell lines and extracellular vesicles (Rishik et al., 2025). A recent comprehensive database, TheMarker contains diverse types of biomarkers used for therapy and monitoring, including miRNAs (Cui et al., 2024; Zhang et al., 2024b). Launched in 2011, miRTarBase a database of experimentally validated MTIs has been manually curated and updated ten times (Cui et al., 2024). In its latest update, miRTarBase extends its scope by integrating miRNA regulatory networks associated with diseases, along with data on miRNA biomarkers, drug resistance, miRNA-targeted small molecule inhibitors, and miRNA oxidation, providing an integrative multidimensional database (Cui et al., 2024). With this update, miRTarBase now features upwards of 3,817 550 validated MTIs from 13,690 studies, representing a notable increase in data volume and improvements in curation workflow (Cui et al., 2024).

The adoption of high-throughput transcriptome sequencing technology, has made the identification of differentially expressed genes in diseases easy, allowing to gain better understanding of disease onset and guiding therapeutic decisions. The use of different bioinformatic analysis approaches, including HMDD and miRtarbase, have provided unique insights into the underlying mechanisms of endometriosis. Based on HMDD, 150 miRNAs have been found associated with endometriosis (Ye et al., 2022). Furthermore, the mechanisms of endometriosis-induced repeated pregnancy loss were discovered to be connected to the PI3K/AKT signaling pathway and platelet activation (Ye et al., 2022). Thus, miRNAs databases seem to be valuable in constructing miRNAs-mRNAs regulatory networks associated with different conditions, allowing precise targeting of the transcriptome and epigenome.

### 3.5 Endometriosis research, still a challenge

While previously cited studies have provided valuable insights, the reproducibility remains a major hindering and limiting factor in endometriosis research. Discrepancies in results need to be treated with caution since the majority of studies have limitations. Study weaknesses include monocentricity of the majority of studies, small sample size, and high heterogeneity in samples collected during different phases of the menstrual cycle. The intra-lesion heterogeneity is an additional limiting factor whose importance is generally underestimated. Study designs overlook key aspects such as (1) endometriosis type and severity; superficial peritoneal endometriosis, ovarian endometrioma and deep infiltrating endometriosis, (2) endometriosis stages; minimal, mild, moderate, and severe, and (3) anatomic distribution of endometriotic lesions; utero sacral ligaments, pouch of Douglas, ovarian fossa. Regarding *in vitro* models, they have severe limitations. Primary cells used in endometriosis research lack purity and are not phenotypically characterized, and cell lines are not genotypically authenticated (Romano et al., 2020). The analysis of sparse and incomplete medical data is a significant challenge in endometriosis research. Complete medical and clinical history, especially hormonal treatment, other illness conditions, past surgical history, and family history of endometriosis, are often missing which introduce biases and affect the generalizability of research findings. Finally, clinical trial landscape in endometriosis is advancing on multiple fronts, with numerous epigenetic focused ongoing trials, namely, micro-RNAs; RC 2.6.2022 (NCT05680350), ADOmiARN (NCT05928442), ENDOmiARN (NCT04728152), FR-21-001 (NCT05244668), STUDY00009584 (NCT05331053), ENDOmiRNA (NCT06414720), EMPOWER (NCT04598698), ENDMET (NCT06168097), 35,617/8/22 (NCT05556213), Pro00009633 (NCT02253251). However, due to epigenetic clinical trials are challenging, no epigenetic blockbuster drug for endometriosis seems to be on the horizon yet. One of the challenges that needs to be overcome is associated with the precise localization and targeted activity of epigenetics-targeted drugs. For instance, histone-modifying enzymes are found both in the nucleus and the cytoplasm. On the other hand, the function of ncRNAs depends on their localization and distribution within intracellular compartments. Hence, a deeper understanding of the intracellular trafficking of epigenetic modifiers is warranted. It is noteworthy that resistance to epigenetic drugs is another limiting factor for epigenetic drug application (Dai et al., 2024). Continued research into the biological and pathological roles of targets for epigenetic drugs is essential.

## 4 Conclusion

Endometriosis is a multifactorial disease involving hormonal, immune, genetic and epigenetic factors that interact in intricate ways to drive endometriosis initiation and progression. Given the heterogeneity inherent in endometriosis, understanding the intricate interplay between these factors could pave the way for developing innovative approaches for accurate diagnosis. Epigenetics plays a central role in the genesis of endometriosis, influencing steroid hormone signaling and modulating the immune microenvironment. The use of non-invasive methods based on epigenetic abnormalities such as DNA methylation, histone modifications and ncRNAs, especially miRNAs and lncRNAs, hold great potential as valuable diagnostic and prognostic biomarkers. Epigenetic biomarkers can further improve timely diagnosis, reduce the cost of diagnosis and treatment, and enhance social wellbeing of women. Unlike DNA methylation, histone modifications still lack a defined mechanism of inheritance and call for more extensive research. Much current focus is on the role played by non-coding RNAs in endometriosis.

Epigenetic research in endometriosis is expected to advance rapidly in the coming years, focusing on developing diagnosis biomarkers and targeted therapies. Much attention must be paid to study designs to avoid non-reproducibility of conclusions from different studies.

## Glossary

AI: Artificial Intelligence
AKT3: AKT serine/threonine kinase 3
BST2: Bone Marrow Stromal Cell Antigen 2
CDK1: Cyclin-Dependent Kinase
CDKN2B: CDKN2B antisense RNA1
ceRNA: Competing endogenous RNA
COX-2: Cyclo-oxygenase 2
CYP17A1: Cytochrome P450 family 17 subfamily A member 1
CYP19A1: Aromatase
DNMT1: DNA methyltransferase 1
DNMT3A: DNA (cytosine-5)-methyltransferase 3 beta
DNMT3B: DNA (cytosine-5)-methyltransferase 3 beta
ER: Estrogen Receptors
ESC: Endometrial Stromal Cell
ESR1: Estrogen receptor α
GATA6: GATA-binding factor 6
GREM2: Gremlin 2, DAN Family BMP Antagonist
HAT: Histone AcetylTransferase
HDAC: Histone Deacetylase
HOXA10: Homeobox A10;
HPGD: 15-hydroxyprostaglandin dehydrogenase
HPV: Human Papillomavirus
HSD3B2: 3b-hydroxysteroid dehydrogenase type 2
IDO1: Indoleamine 2, 3- dioxygenase
IER3: Immediate Early Response 3
IL-10: Interleukin-10
KIR3DX1: Killer Cell Immunoglobulin Like Receptor, Three Ig Domains X1
LIF: Leukaemia inhibitory factor
lncRNA: Long non-coding RNAs
miRNA: microRNA
ML: Machine Learning
mRNA: Messenger RNAs
NF-IL6: Nuclear Factor for Interleukin-6 expression
NGS: Next-Generation Sequencing
NoEM: Normal endometrial stromal cell
NR5A1: Nuclear receptor subfamily 5, group A, member 1
PCNA: Proliferating Cell Nuclear Antigen
PGE2: Prostaglandin E2
PGR: Progesterone Receptor
PIK3CG: Phosphatidylinositol-4,5- bisphosphate 3-kinase catalytic subunit gamma
piRNA: PIWI-interacting RNAs
PR-B: Progesterone Receptor B
qPCR: Quantitative Polymerase Chain Reaction
RNASE1: Ribonuclease A family member 1, pancreatic
SCC: Side-Chain Cleavage
SF-1: Steroidogenic Factor 1
SFN: Stratifin
siRNA: small interfering RNA
StAR: Steroidogenic Regulatory Protein
TGF-β: Transforming Growth Factor-beta
TMEM184A: Transmembrane Protein 184A.
