# Supplementary material for: Leveraging epigenetic aberrations in the pathogenesis of endometriosis: from DNA methylation to non-coding RNAs
Source: Front Genet. 2025 Jul 28;16:1597287. doi: 10.3389/fgene.2025.1597287 (PMC12336057; doi:10.3389/fgene.2025.1597287)
Supplement: Supplementary file 1 [file Table6.docx]

**Table 6**. MicroRNAs and long non-coding RNAs reported in the immune microenvironment of endometriosis.

| Dysregulated microRNAs | References | Dysregulated long non-coding RNAs | References |
| --- | --- | --- | --- |
| Downregulation of miR-199a | (Dai et al., 2012) | Downregulation of H19 | (Liu et al., 2019) |
| Downregulation of miR-16 | (Wang et al., 2020) | Upregulation of MALAT-1 | (Tan et al., 2022) |
| Downregulation of miR-182 | (Wu and Zhang, 2021) | Upregulation of LINC00339 | (Holdsworth-Carson et al., 2021) |
| Upregulation of miR-20a | (Lin et al., 2012) | Upregulation of HOTAIR | (Zhang et al., 2022) |
| Downregulation of miR-26b-5p, miR-215-5p, instead of upregulation of miR-6795-3p | (Wu et al., 2022) | Upregulation of LINC01018 | (Jiang et al., 2020) |
| Downregulation of miR-17 | (Wang et al., 2018) | Upregulation of LINC01272 | (Jiang et al., 2020) |
| Upregulation of miR-302a | (Lin et al., 2014) | Upregulation of CHL1-AS1 | (Liu et al., 2021) |
| Upregulation of miR-301a-3p | (Huang et al., 2022b) | Upregulation of AFAP1-AS1 | (Bi et al., 2021) |
| Upregulation of miR-146b | (Zhang et al., 2019) | Upregulation of MIR202HG | (Bi et al., 2021) |
| Upregulation of miR-887-5p | (Huang et al., 2022a) | Upregulation of LINC02381 | (Yin et al., 2022) |
| Upregulation of miR-22-3p | (Zhang et al., 2020) | Upregulation of LINC01140, MSC-AS1, HAGLR, CKMT2-AS1, JAKMIP2-AS1, and AL365361.1 | (Gu et al., 2021) |

References

Bi, J., Wang, D., Cui, L., and Yang, Q. (2021). RNA sequencing-based long non-coding RNA analysis and immunoassay in ovarian endometriosis. *Am. J. Reprod. Immunol.* 85, e13359. doi: 10.1111/AJI.13359

Dai, L., Gu, L., and Di, W. (2012). MiR-199a attenuates endometrial stromal cell invasiveness through suppression of the IKKβ/NF-κB pathway and reduced interleukin-8 expression. *Mol. Hum. Reprod.* 18, 136–145. doi: 10.1093/MOLEHR/GAR066

Gu, C., Meng, Y., Meng, Q., Fan, W., Ye, M., Zhang, Q., et al. (2021). Exploring the Potential Key IncRNAs with Endometriosis by Construction of a ceRNA Network. *Int. J. Gen. Med.* 14, 4161–4170. doi: 10.2147/IJGM.S321648

Holdsworth-Carson, S. J., Churchill, M., Donoghue, J. F., Mortlock, S., Fung, J. N., Sloggett, C., et al. (2021). Elucidating the role of long intergenic non-coding RNA 339 in human endometrium and endometriosis. *Mol. Hum. Reprod.* 27. doi: 10.1093/MOLEHR/GAAB010

Huang, Y., Yan, S., Dong, X., Jiao, X., Wang, S., Li, D., et al. (2022a). Deficiency of MST1 in endometriosis related peritoneal macrophages promoted the autophagy of ectopic endometrial stromal cells by IL-10. *Front. Immunol.* 13, 993788. doi: 10.3389/FIMMU.2022.993788/BIBTEX

Huang, Y., Zhu, L., Li, H., Ye, J., Lin, N., Chen, M., et al. (2022b). Endometriosis derived exosomal miR-301a-3p mediates macrophage polarization via regulating PTEN-PI3K axis. *Biomed. Pharmacother.* 147, 112680. doi: 10.1016/J.BIOPHA.2022.112680

Jiang, L., Zhang, M., Wang, S., Xiao, Y., Wu, J., Zhou, Y., et al. (2020). LINC01018 and SMIM25 sponged miR-182-5p in endometriosis revealed by the ceRNA network construction. *Int. J. Immunopathol. Pharmacol.* 34. doi: 10.1177/2058738420976309/SUPPL_FILE/SJ-XLSX-2-IJI-10.1177_2058738420976309.XLSX

Lin, S. C., Li, Y. H., Wu, M. H., Chang, Y. F., Lee, D. K., Tsai, S. Y., et al. (2014). Suppression of COUP-TFII by Proinflammatory Cytokines Contributes to the Pathogenesis of Endometriosis. *J. Clin. Endocrinol. Metab.* 99, E427. doi: 10.1210/JC.2013-3717

Lin, S. C., Wang, C. C., Wu, M. H., Yang, S. H., Li, Y. H., and Tsai, S. J. (2012). Hypoxia-Induced MicroRNA-20a Expression Increases ERK Phosphorylation and Angiogenic Gene Expression in Endometriotic Stromal Cells. *J. Clin. Endocrinol. Metab.* 97, E1515–E1523. doi: 10.1210/JC.2012-1450

Liu, T., Liu, M., Zheng, C., Zhang, D., Li, M., and Zhang, L. (2021). <p>Exosomal lncRNA CHL1-AS1 Derived from Peritoneal Macrophages Promotes the Progression of Endometriosis via the miR-610/MDM2 Axis</p>. *Int. J. Nanomedicine* 16, 5451–5464. doi: 10.2147/IJN.S323671

Liu, Z., Liu, L., Zhong, Y., Cai, M., Gao, J., Tan, C., et al. (2019). LncRNA H19 over-expression inhibited Th17 cell differentiation to relieve endometriosis through miR-342-3p/IER3 pathway. *Cell Biosci.* 9. doi: 10.1186/S13578-019-0346-3

Tan, K., Mo, H., Guo, L., and Wang, B. (2022). MALAT1 accelerates proliferation and inflammation and suppresses apoptosis of endometrial stromal cells via the microRNA-142-3p/CXCR7 axis. *Reprod. Biol.* 22, 100675. doi: 10.1016/J.REPBIO.2022.100675

Wang, F., Wang, H., Jin, D., and Zhang, Y. (2018). Serum miR-17, IL-4, and IL-6 levels for diagnosis of endometriosis. *Med. (United States)* 97. doi: 10.1097/MD.0000000000010853

Wang, X., Ren, R., Shao, M., and Lan, J. (2020). MicroRNA-16 inhibits endometrial stromal cell migration and invasion through suppression of the inhibitor of nuclear factor-κB kinase subunit β/nuclear factor-κB pathway. *Int. J. Mol. Med.* 46, 740–750. doi: 10.3892/IJMM.2020.4620/HTML

Wu, M., and Zhang, Y. (2021). MiR-182 inhibits proliferation, migration, invasion and inflammation of endometrial stromal cells through deactivation of NF-κB signaling pathway in endometriosis. *Mol. Cell. Biochem.* 476, 1575–1588. doi: 10.1007/S11010-020-03986-2/METRICS

Wu, Y., Yuan, W., Ding, H., and Wu, X. (2022). Serum exosomal miRNA from endometriosis patients correlates with disease severity. *Arch. Gynecol. Obstet.* 305, 117. doi: 10.1007/S00404-021-06227-Z

Yin, M., Zhai, L., Wang, J., Yu, Q., Li, T., Xu, X., et al. (2022). Comprehensive Analysis of RNA-Seq in Endometriosis Reveals Competing Endogenous RNA Network Composed of circRNA, lncRNA and mRNA. *Front. Genet.* 13, 828238. doi: 10.3389/FGENE.2022.828238/BIBTEX

Zhang, L., Li, H. H., Yuan, M., Li, D., and Wang, G. Y. (2020). Exosomal miR-22-3p derived from peritoneal macrophages enhances proliferation, migration, and invasion of ectopic endometrial stromal cells through regulation of the SIRT1/NF-κB signaling pathway. *Eur. Rev. Med. Pharmacol. Sci.* 24, 571–580. doi: 10.26355/EURREV_202001_20033

Zhang, L., Yu, Z., Qu, Q., Li, X., Lu, X., and Zhang, H. (2022). <p>Exosomal lncRNA HOTAIR Promotes the Progression and Angiogenesis of Endometriosis via the miR-761/HDAC1 Axis and Activation of STAT3-Mediated Inflammation</p>. *Int. J. Nanomedicine* 17, 1155–1170. doi: 10.2147/IJN.S354314

Zhang, Z., Li, H., Zhao, Z., Gao, B., Meng, L., and Feng, X. (2019). miR-146b level and variants is associated with endometriosis related macrophages phenotype and plays a pivotal role in the endometriotic pain symptom. *Taiwan. J. Obstet. Gynecol.* 58, 401–408. doi: 10.1016/J.TJOG.2018.12.003
